# Identification and bioinformatic characterization of a serum miRNA signature for early detection of laryngeal squamous cell carcinoma

**DOI:** 10.1186/s12967-024-05385-3

**Published:** 2024-07-10

**Authors:** Michela Falco, Chiara Tammaro, Alessia Maria Cossu, Takashi Takeuchi, Rossella Tufano, Michele Ceccarelli, Giuseppe Scafuro, Silvia Zappavigna, Anna Grimaldi, Marianna Scrima, Alessandro Ottaiano, Giovanni Savarese, Antonio Fico, Massimo Mesolella, Morena Fasano, Giovanni Motta, Eva Aurora Massimilla, Raffaele Addeo, Filippo Ricciardiello, Michele Caraglia, Gabriella Misso

**Affiliations:** 1 Laboratory of Molecular and Precision Oncology, BIOGEM Scarl, Institute of Genetic Research, 83031 Ariano Irpino, Italy; 2 Department of Precision Medicine, University of Campania “Luigi Vanvitelli”, 80138 Naples, Italy; 3 Molecular Diagnostics Division, Wakunaga Pharmaceutical Co., Ltd, Hiroshima, 739-1195 Japan; 4 Laboratory of Bioinformatics and Computational Biology, BIOGEM Institute of Molecular Biology and Genetics, 83031 Ariano Irpino, Italy; 5 U.P. Cytometric and Mutational Diagnostics, AOU Policlinico, University of Campania “Luigi Vanvitelli”, Via Luciano Armanni 5, 83031 Naples, Italy; 6 Istituto Nazionale Tumori Di Napoli, IRCCS “G. Pascale”, Via M. Semmola, 80131 Naples, Italy; 7 AMES, Centro Polidiagnostico Strumentale, 80013 Naples, Italy; 8 Unit of Otorhinolaryngology, Department of Neuroscience, Reproductive Sciences and Dentistry, Federico II University of Naples, Via Gaetano Filangieri, 36, 80131 Naples, Italy; 9 ENT Department, L. Vanvitelli University, 80131 Naples, Italy; 10 Oncology Operative Unit, Hospital of Frattamaggiore, ASLNA-2NORD, 80020 Naples, Italy; 11 Division of Otorhinolaryngology, “A. Cardarelli” Hospital, 80131 Naples, Italy

**Keywords:** Laryngeal Squamous Cell Cancer, miRNA, miRNA signature, ROC curve, miR-532, miR-93, miR-223, Overall survival, Cancer, Gene targets

## Abstract

**Background:**

The growing understanding of cancer biology and the establishment of new treatment modalities has not yielded the expected results in terms of survival for Laryngeal Squamous Cell Cancer (LSCC). Early diagnosis, as well as prompt identification of patients with high risk of relapse would ensure greater chance of therapeutic success. However, this goal remains a challenge due to the absence of specific biomarkers for this neoplasm.

**Methods:**

Serum samples from 45 LSCC patients and 23 healthy donors were collected for miRNA expression profiling by TaqMan Array analysis. Additional 20 patients and 42 healthy volunteers were included for the validation set, reaching an equal number of clinical samples for each group. The potential diagnostic ability of the such identified three-miRNA signature was confirmed by ROC analysis. Moreover, each miRNA was analyzed for the possible correlation with HNSCC patients’ survival and TNM status by online databases Kaplan–Meier (KM) plotter and OncomiR. In silico analysis of common candidate targets and their network relevance to predict shared biological functions was finally performed by PANTHER and GeneMANIA software.

**Results:**

We characterized serum miRNA profile of LSCC patients identifying a novel molecular signature, including miR-223, miR-93 and miR-532, as circulating marker endowed with high selectivity and specificity. The oncogenic effect and the prognostic significance of each miRNA was investigated by bioinformatic analysis, denoting significant correlation with OS. To analyse the molecular basis underlying the pro-tumorigenic role of the signature, we focused on the simultaneously regulated gene targets—IL6ST, GTDC1, MAP1B, CPEB3, PRKACB, NFIB, PURB, ATP2B1, ZNF148, PSD3, TBC1D15, PURA, KLF12—found by prediction tools and deepened for their functional role by pathway enrichment analysis. The results showed the involvement of 7 different biological processes, among which inflammation, proliferation, migration, apoptosis and angiogenesis.

**Conclusions:**

In conclusion, we have identified a possible miRNA signature for early LSCC diagnosis and we assumed that miR-93, miR-223 and miR-532 could orchestrate the regulation of multiple cancer-related processes. These findings encourage the possibility to deepen the molecular mechanisms underlying their oncogenic role, for the desirable development of novel therapeutic opportunities based on the use of short single-stranded oligonucleotides acting as non-coding RNA antagonists in cancer.

**Supplementary Information:**

The online version contains supplementary material available at 10.1186/s12967-024-05385-3.

## Introduction

Laryngeal Squamous Cell Cancer (LSCC) is the sixth most common cancer worldwide. The vast majority of laryngeal cancers are well-differentiated squamous cell carcinomas (LSCCs), accounting over 90% of the cases [1]. A minority of cases represent squamous cell variants. The histopathological range of the SCCs varies significantly from hyperplasia, dysplasia and carcinoma in situ to invasive cancer and involves different subsites of the larynx, with distinct implications in symptomatic manifestations, spreading patterns and treatment options. The landscape of metastatic progression usually depends on primary mass location and on lymph node involvement. The latter is a hallmark of supraglottic cancers for almost 55% patients. On the other hand, vocal cords do not present lymphatic involvement risk [2, 3]. The 5-years relative survival rate for patients diagnosed for LSCC is 60.7%, mainly due to the high percentage of diagnosis at advanced stage, which negatively impact on prognosis and mortality [4]. Additionally, it was demonstrated that the incidence and prevalence have increased by 12.0% and 23.8% during past three decades, respectively. On the other hand, mortality has declined by approximately 5.0%. Tabacco and alcohol consumption are the most important risk factors in LSCC pathogenesis [5], prompting a multiplicative effect on disease development [6]. Environmental pollution, Human Papilloma Virus (HPV) infection, gastroesophageal reflux disease or exposure to asbestos are additional risk factors.

Cancer patients with similar clinical and pathological parameters can often have different prognosis [7]. LSCC carcinogenesis has been widely investigated in the last years, focusing on genetic and molecular modifications occurring in neoplastic transformation and progression. Many studies are focused on proteins, genes or chromosomes, whose aberrations in neoplastic tissues could correlate with the biological behaviour of disease such as: pro-oncogenes, growth factors and onco-suppressive genes [8]. The main pathological pathways implicated in LSCC tumorigenesis include the dysregulation of cellular survival and proliferation (Tp53 and EGFR), cell-cycle control (CDKN1A) and cellular differentiation (NOTCH1) [9, 10] mediators. In the last 20 years, epithelial–mesenchymal transition EMT has been increasingly studied, highlighting the main difference between migration of normal keratinocytes and transformed epithelial cells. This process regards exclusively cancer cells, and it is characterised by adherents’ junctions breaking and epithelial markers’ (*i.e.* cytokeratins and E-cadherin) reduced levels, accompanied by mesenchymal markers’ (*i.e.* fibronectin, N-cadherin, and Vimentin) upregulation [11]. Recently it has been demonstrated the correlation between this process and IL6 pro-inflammatory cytokine activation in several tumors especially in LSCC [12].

Biopsy and imaging examination represent the conventional gold standard for cancer detection. However, these tools have low sensitivity and the identification of new non-invasive and highly sensitive biomarkers are strongly required in order to increase the survival of these patients [13].

In recent years, the discovery of microRNA (miRNAs) in biological fluids has generated great interest for their potential use as biomarkers, given their modulation associated with specific biological/pathological conditions [14]. In fact, miRNAs are routinely secreted by cancer cells into biological fluids, such as serum, plasma, saliva, urine and breast milk [15, 16]. They can impact not only in the tissue of origin but also in the regulation of gene expression of distant target cells [17]. Circulating miRNAs are released into bloodstream in many forms and the reasons for their high stability remain largely unknown, although many hypotheses have been proposed [18]: (i) circulating miRNAs may have unique modifications, such as methylation, adenylation, and uridylation, that increase their stability protecting them against RNAses [19]; (ii) they are protected by encapsulation in cell-derived macrovesicles [20] or (iii) through specific RNA-binding proteins and lipoproteins [21, 22].

Despite their origin and release have not been fully elucidated, three different mechanisms are suggested [18]: (i) passive release from apoptotic or necrotic cells, as well as from damaged tissues, or from cells with a short half-life, such as platelets; (ii) active secretion through cell-derived macrovesicles, including exosomes and shedding vesicles; [18] (iii) active cellular secretion of both free and RNA binding protein-complexed miRNAs. The latter are represented by nucleophosmin (the nucleolar phosphoprotein NMP1) [18], high/low density lipoproteins [23] and Argonaute protein family (AGO) [20, 21, 24].

Circulating miRNAs have many of the essential characteristics of good biomarkers: they are stable in circulation, resistant to RNase digestion, extreme pH, high temperatures, prolonged storage and multiple freeze–thaw cycles [25, 26]. Moreover, circulating miRNA represent an excellent target for the nanostructured biosensors increasingly developed and exploited in the diagnostic field [27]. Nevertheless, the clinical efficacy of miRNAs as circulating biomarkers can be affected by a number of variables such as sample collection and processing, RNA extraction efficiency, as well as other technical aspects involved in the success of qRT-PCR and data analysis; however, correct operations are strictly required in order to increase sensitivity and to obtain reliable results [28].

In the last years, the use of increasingly sophisticated techniques, such as low-density arrays, has enabled the detection of several differentially expressed miRNAs in body fluids of cancer patients compared to healthy volunteers, demonstrating the possibility to identify miRNA signatures representative of specific diseases. The profiling of circulating miRNAs is extremely promising as minimally invasive diagnostic and prognostic tool for various cancers including lung, prostate, breast, colon, gastric, cervical, head and neck cancers [13, 29].

The current study aimed to define a serum miRNA signature to enable LSCC diagnosis at early stage and prognosis prediction, as well as to analyse the putative functional role of the identified miRNAs through an accurate bioinformatic analysis. Preliminary in vitro experimental data were also provided to explore the impact of miR-223 overexpression on cell viability.

## Materials and methods

### Clinical samples

Blood samples were collected from LSCC patients and healthy donors, enrolled at the Ear, Nose and Throat Division of the University of Naples Federico II, the University of Campania L.Vanvitelli, Monaldi and Antonio Cardarelli Hospitals. The study was in accordance with the Institutional Ethics Committee guidelines, Italian law and the Declaration of Helsinki, and was approved by the Ethics Committee of University of Campania “Luigi Vanvitelli”—Azienda Ospedaliera Universitaria “Luigi Vanvitelli”—AORN “Ospedali dei Colli” (Approval number: 25445/2021). Informed consent was obtained from all patients. The serum collection tube including blood was centrifuged at 3000 rpm, at room temperature (R.T.) for 5 min. The supernatant was collected as serum into a 2 mL cryotube in draft chamber and stocked in – 80 ℃ freezer until the extraction of miRNA.

Forty-five LSCC sera from 23 patients suffering from lymph node metastases (N^+^), 22 patients without lymph node involvement (N^─^), and 23 sera samples collected from healthy donors were enrolled for the Microarray study. 65 LSCC blood samples (37 N and 28 N^+^) and 65 normal blood samples, including all subjects already joined in the preliminary screening, were used for the validation set.

### Cell culture and in vitro transfection of LSCC cells

Cell lines were purchased from CLS Cell Lines Service (Eppelheim, Germany) and ATCC. Immortalized human keratinocytes (HaCaT) and HNO-210 cell lines were grown in DMEM (MICROGEM DMEM Cat# AL066A), while HEp-2 and FaDu lines were grown in Minimum Essential Media (MEM) (ATTC Cat# 30–2003). Cell line culture media were supplemented with 10% fetal bovine serum (FBS) (MICROGEM, Naples, Italy), 50 U/mL penicillin, 500 mg/mL streptomycin, and 4 mM glutamine (Gibco, Life Technologies, Carlsbad, CA) in a humidified atmosphere with 5% CO2 at 37 ℃.

The LSCC HNO-210 cells were placed at a density of 4.0 × 10^5^ cells per well in Opti-MEM media (Gibco, Life Technologies, Carlsbad, CA, USA) in a six-well plate. After incubation for 24 h, the cells were transfected with mimic miR-223 (mirVana miRNA mimic) (Life Technologies, Carlsbad, CA, USA), or scramble negative control (mirVana miRNA mimic, negative control #1) (Life Technologies, Carlsbad, CA, USA), or antago-miR-223 (mirVana miRNA inhibitor) (Life Technologies, Carlsbad, CA, USA), or scramble negative control (mirVana miRNA inhibitor, negative control #1) (Life Technologies, Carlsbad, CA, USA) using Lipofectamine 2000 (Life Technologies, Carlsbad, CA, USA), according to the provided instructions. After incubating the transfected cells in culture for 24 h, the medium was replaced into fresh complete medium.

### RNA extraction

Serum miRNA isolation was carried out using miRNeasy Serum/Plasma Kit (QIAGEN), according to the manufacturer’s manual. In this extraction procedure, 3.5 µL of 1 nM cel-miR-39-3p mimic (mirVana^®^ miRNA mimics, Ambion) was added into 200 µL of serum as an external control. Total RNA, including miRNA, was extracted from cultured cells using a mirVana PARIS kit (Ambion, Life Technologies, Carlsbad, CA, USA) according to the manufacturer’s protocol. The integrity, quality (Abs. 260 nm/230 nm and 260 nm/280 nm) and quantity of total RNA were assessed by the NanoDrop ND-1000 Spectrophotometer (Thermo Scientific, Wilmington, DE, USA). The purified serum miRNAs and RNA samples from cells were stored in − 80 ℃ freezer until further processing.

### MTT Assay

Transfected HNO-210 cells were seeded into 96-well plates at 2,000 cells/well and incubated at 37 ℃ in 5% CO2. After 24, 48 and 72 h, the cells were stained using MTT reagent (Sigma-Aldrich, St. Louis, MO, USA) for 4 h. Acidified isopropanol was added to dissolve MTT into each well and mixed for 20 min by shaking. Absorbance at 570 nm was measured by an iMark microplate absorbance reader (Bio-Rad, Hercules, CA, USA).

### Microarray screening assay

Serum miRNAs purified from 45 LSCC patients and 23 healthy donors were enrolled for miRNAs screening assay. The specimens of LSCC cohort were divided in two groups, *i.e.* 22 lymph node metastases positive (N^+^) and 23 negative (N^─^) serum samples. For reverse transcription, an equal amount of serum miRNAs from different patients or donors, were mixed into 5 pools for each group (Table 1**)**.

**Table 1:** Number of samples from LSCC patients or healthy donors for each pool used in miRNAs screening

Pool	*LSCC patients N*^+^ *N*^*─*^		*Healthy donors*
1	5	5	5
2	5	5	5
3	4	5	5
4	4	4	4
5	4	4	4

Starting from each RNA pool, cDNA was synthesized with Megaplex RT Primers, Human Pool A v2.1 (Applied Biosystems, California, USA) and TaqMan MicroRNA Reverse Transcription Kit (Applied Biosystems, California, USA), according to manufacturer’s instructions. Human Pool A v2.1 contains RT primers for 377 unique microRNAs and 4 controls. Subsequently, cDNA was pre-amplified with Megaplex PreAmp Primers Human Pool A v2.1 (Applied Biosystems, California, USA) and TaqMan PreAmp Master Mix (Applied Biosystems, California, USA).

The miRNA expression profiling was finally performed using TaqMan Array Human MicroRNA A Cards v2.0 (Applied Biosystems, California, USA) and TaqMan^®^ Universal PCR Master Mix (Applied Biosystems, California, USA), following manufacturer’s manuals. The assay was run on Viia 7 real-time PCR system (Applied Biosystems, California, USA) as previously described [30].

The Ct value was determined using Viia7 software (Applied Biosystems, California, USA) and setting a threshold of 0.2. For normalization of miRNAs expression, NormFinder analysis was conducted with corresponding R software available online (http://moma.dk/normfinder-software), and miR-222, which was the most stably expressed among all pools, was selected as a reference miRNA. Thus, ΔCt was obtained using the formula: ΔCt = Ct target miRNA—Ct miR-222, and the relative miRNA expression was calculated with the ΔΔCt method (ΔΔCt = ΔCt interest group—ΔCt control group). Fold change was calculated with the Eq. 2^−ΔΔCt^ method and converted to logarithm (Log2).

### Real-time quantitative PCR

For validation of miRNA candidates, miRNAs samples, originated from sera of both LSCC patients and healthy donors, and from LSCC and normal cell lines, were tested by qRT-PCR. cDNA synthesis was performed with TaqMan MicroRNA Reverse Transcription Kit (Applied Biosystems, California, USA), according to manufacturer’s manual. In order to detect miR-223, miR-93 and miR-532 in sera samples and basal expression levels of miR-223 in cell lines quantitative PCR was performed using TaqMan Fast Universal PCR Master Mix (2X) no AmpErase UNG (Applied Biosystems, California, USA) under the control of ViiA 7 Real-Time PCR System (Applied Biosystems, California, USA). Comparative real-time PCR (RT-PCR) was performed in triplicate, including no template controls, and relative expression was calculated using the comparative cross-threshold (Ct) method.

Cycle threshold (Ct) value was calculated using ViiA^™^ 7 Software (Applied Biosystems) with threshold set to 0.2. Subsequently, for normalization of target gene expression level, ΔCt was derived by formula: Ct of target gene − Ct of reference gene; reference gene for the evaluation of miRNA candidates were U6 snRNA for cell lines and cel-miR-39-3p (exogenous miRNA reference) for sera samples. ΔΔCt was calculated by formula: ΔCt of interest group—ΔCt of control group, and then 2^−ΔΔCt^ was derived as a fold change (FC) of target gene expression.

### In silico bioinformatic analysis

#### Survival analysis

Impact of the three selected serum miRNAs (miR-93, miR-223, miR-532) expression in HNSCC patients was determined to identify prognostic risk factor. In detail, we used an integrated online bioinformatic tool, Kaplan–Meier Plotter (http://kmplot.com), which includes both clinical and expression data, and allowed to perform a Kaplan–Meier survival analysis to validate the prognostic value of a set of previously published survival-associated miRNAs. The experimental parameters set for the curve analysis of each miRNA were the following: patients were splitted by”Auto select best cutoff”; “compute median survival” and “censore at threshold” were flagged, considering a median follow up of 240 months. Moreover, to obtain more specific results we also analyzed the OS of HNSCC patients overexpressing miR-223, miR-93 and miR-532, with respect to stage, gender and grade. In this case we used the same experimental parameters, and we restricted analysis to the cited subtypes by selecting the items of interest.

#### Gene targets identification

The publicly available algorithm miRTargetlink allowed to perform an in-silico analysis of common target candidates between the selected miR-223-3p, miR-532-5p and miR-93-5p. miRTargetLink 2.0 is a novel version of an interactive tool for systems biology applications that provide a large set of miRNA gene associations from published repositories [miRTarBase, mirDIP, miRDB and miRATBase] and extend it by the pathway data from the recent release of miRPathDB 2.0.

#### Enrichment analysis of common target candidates

PANTHER (Protein ANalysis THrough Evolutionary Relationships) (v16.0) was used for miRNA pathways enrichment analysis. PANTHER server can analyse gene lists, and expression data files; map lists to multiple annotation data sources from PANTHER and the Gene Ontology Consortium, as well as biological pathway; overlay results on pathway diagrams to visualize the relationships between genes/proteins in known pathways.

Reactome is a curated database of pathways and reactions in human biology also queried to deepen the overrepresented mechanisms. Reactome content frequently cross-references other resources e.g. NCBI, Ensembl, UniProt, KEGG (Gene and Compound), ChEBI, PubMed and GO (https://reactome.org/).

#### Co-regulated network construction and topology analysis

The GeneMANIA prediction server (http://genemania.org) is a flexible web site for analysing gene lists and generating hypotheses about gene function. We used GeneMANIA to select specific sets of networks and connections among query genes.

#### Dysregulated miRNAs and their association with HNSCC pathogenesis

For cancer, the analysis of miRNA expression has proved to be extremely useful for the identification of genes, capable of determining the pathological state and for the development of new hypotheses on physiology useful for answering diagnostic, prognostic and functional questions. We used the online resource OncomiR, for exploring miRNA dysregulation in cancer. Using combined miRNA-seq, RNA-seq and clinical data from The Cancer Genome Atlas, the software performed statistical analyses to identify dysregulated miRNAs that are associated with tumor development, progression, and clinical parameters in HNSCC; the possibility of taking these relationships into consideration may therefore be relevant for identifying the biomarker genes that characterize the disease occurrence.

### Statistical analysis

Construction of clustered heatmap for microarray analysis was performed using heatmap.2 function of gplots package in statistical analysis tool R (version 3.4.3). To evaluate expression difference between two groups, student’s t-test was used for calculating p-value. To proceed with the validation, sample size was calculated by statistical analysis using G*Power 3.1.9.7 software. Sufficient sample size was estimated to be at least 59 sera samples for each experimental group taken into consideration with α value of 0.05 (5% maximum allowed risk), to lower the type I error and power of 0.85 (85%) to increase the accuracy and statistical validity of analysis.

Graphs were obtained using GraphPad Prism (version 7.00) and significant differences were determined at *p* ≤ *0.05* according to Student’s t test.

In receiver operating characteristic (ROC) curve analysis, area under the curve (AUC) was calculated to determine cut off and further calculate sensitivity, specificity, and accuracy for investigation of diagnostic performance (R version 3.4.3). The clustering analysis between miRNA expression levels and clinical characteristics was performed by R (version 3.4.3). To examine relation between miRNA expression levels and clinical features, correlation coefficient and p-value were calculated by GraphPad Prism (version 7.00).

## Results

### Definition of serum miRNA signature and its diagnostic value in LSCC

**Fig. 1:**
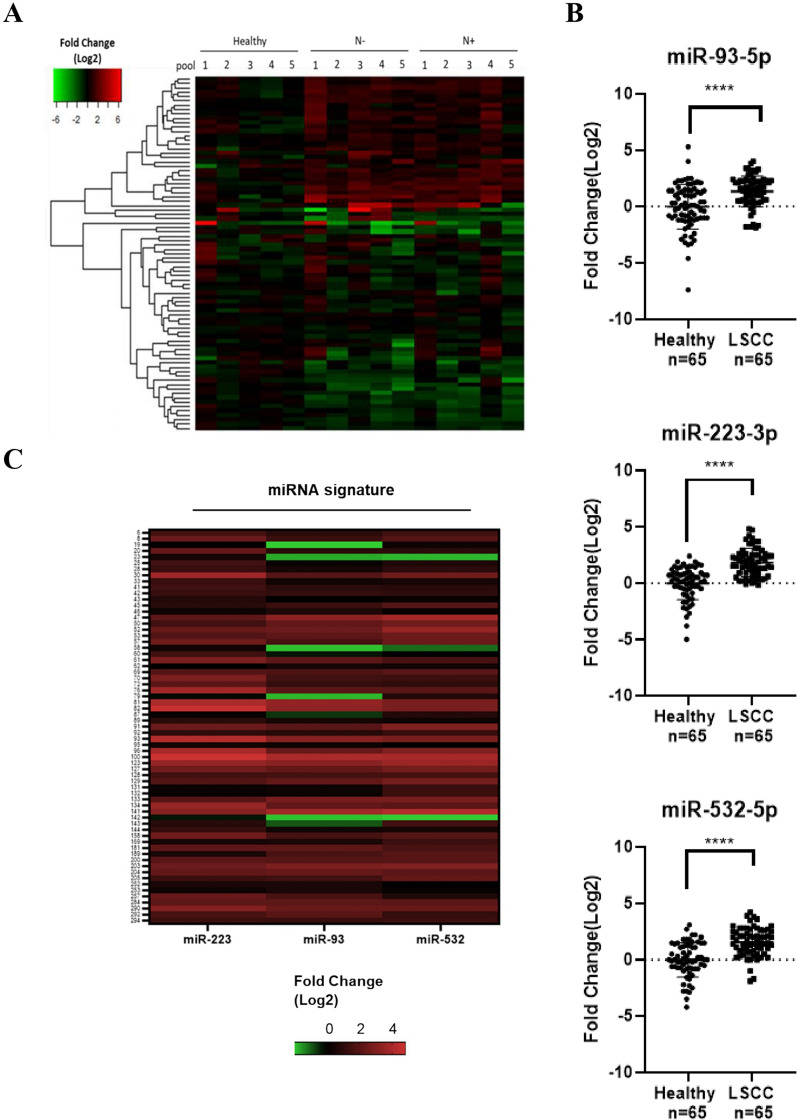
**A** Serum miRNAs global expression pattern among N^+^ or N^─^ LSCC samples compared to healthy donor groups. The hierarchical clustering heatmap summarizes the 81 detectable serum miRNA expression levels, normalized by miR-222 as endogenous control. **B** Serum miR-93, miR-223, and miR-532 expression levels in LSCC patients. The expression levels of serum miR-93, miR-223, and miR-532 in LSCC patients (n = 65) compared with healthy donors (n = 65), validated by qRT-PCR. Exogenous cel-miR-39 was used as normalizer. The p-value was calculated by t-test ****p < 0.001. **C** Serum miR-93, miR-223, and miR-532 expression levels for each of 65 LSCC patients. The heatmap shows the miRNA expression levels, normalized on the exogenous cel-miR-39, for each individual patient

We investigated serum miRNA expression profile perturbation in LSCC patients to determine new reliable non-invasive biomarker candidates for the definition of LSCC diagnosis and prognosis, as well as for the identification of new therapeutic targets. A high-throughput PCR array analysis was performed following miRNA extraction from a cohort of 45 LSCC patients. Among these, 22 patients had lymph node metastases (N^+^) and the remaining 23 showed no preoperative evidence of lymph nodal involvement (N^─^). Twenty-three healthy donors were also included as reference control. Using TaqMan Array Human MicroRNA A Cards v2.0 (Applied Biosystems) we evaluated the simultaneous expression of 377 mature miRNAs; the differential expression patterns of the 81 detected miRNAs were represented with a hierarchical clustering heatmap (Fig. 1A).

The statistical analysis performed on the obtained dataset showed significant modulation patterns among the groups. In details, 3 inclusion criteria have been established: (i) Mean Ct < 32.0, (ii) Mean Fold change (Log2) < -1.0 or > 1.0, (iii) *p*-value < 0.05, to select reliable miRNAs dysregulated from the 81 candidates. Applying these criteria, 11 up- and 5 down-regulated miRNAs emerged in the comparison between cancer and healthy groups, while no significant differences were reported between N^+^ and N^─^ groups (Table 2).

**Table 2:** Differential expression of serum miRNAs in LSCC patients

microRNA	Regulation	Fold change (Log 2)	S.D	95% CI	*p-value*	*q- value*
miR-532	Up	2.1	0.7	1.7–2.5	0.001	0.014
miR-93	Up	1.8	0.4	1.5–2.1	0.000	0.014
miR-451	Up	1.5	1.1	0.8–2.1	0.021	0.187
miR-140	Up	1.4	0.4	1.1–1.6	0.001	0.014
miR-223	Up	1.3	0.4	1.1–1.6	0.000	0.014
miR-16	Up	1.3	0.4	1.0–1.6	0.001	0.014
miR-20b	Up	1.3	0.5	1.0–1.6	0.001	0.090
miR-29a	Up	1.3	0.5	1.0–1.5	0.001	0.090
miR-132	Up	1.1	0.9	0.6–1.7	0.030	0.253
miR-25	Up	1.0	0.6	0.7–1.4	0.011	0.187
miR-20a	Up	1.0	0.8	0.5–1.5	0.039	0.253
miR-95	Down	− 1.0	1.0	− 1.6–− 0.4	0.050	0.253
miR-150	Down	− 1.1	0.4	− 1.4–− 0.9	0.001	0.014
miR-891a	Down	− 1.1	0.8	− 1.6–− 0.7	0.013	0.187
miR-331	Down	− 1.2	0.6	− 1.6–− 0.9	0.001	0.090
MIR-374–374	Down	− 1.4	1.0	− 2.0–− 0.8	0.013	0.187

Based on the fold change values and on their statistical significance, we focused on 3 up-regulated miRNAs—miR-93, miR-223, and miR-532—as possible diagnostic marker candidates to be validated in a larger population. For the validation set we included additional 20 patients to the 45 previously enrolled in the screening set, for a total amount of 65 LSCC patients (37N^─^ and 28 N^+^). An equal number of healthy volunteers was included. In Table 3 we summarized the clinic-pathological features of both discovery and validation cohorts.

**Table 3:** Clinical characteristics of LSCC patients

Clinical Features	Discovery cohort	Validation cohort
Number of patients	45	65
Mean age (years ± S.D.)	60.1 ± 9.8	61.8 ± 8.7
≤ 60	23 (51.1)	30 (46.2)
> 60	22 (48.9)	35 (53.8)
Gender		
Male	42 (93.3)	56 (86.2)
Female	3 (6.7)	9 (13.8)
T classification		
T1-T2	9 (20)	23 (35.4)
T3-T4	36 (80)	42 (64.6)
Lymph node metastasis		
Negative	23 (51.1)	37 (56.9)
Positive	22 (48.9)	28 (43.1)

By RT-qPCR analysis we confirmed the significant up-regulation of miR-93 (Median fold change = 2.8, *p*-value = 7.4 × 10^–6^), miR-223 (Median fold change = 3.3, *p*-value = 1.8 × 10^–12^), and miR-532 (Median fold change = 3.1, *p*-value = 9.7 × 10^–10^) in cancer group compared to the healthy one, consistently with the discovery phase (Fig. 1B).

Furthermore, we examined the modulation trend of the three miRNAs for each individual patient (Fig. 1C), observing that 58.5% of patients showed a simultaneous up-regulation (fold change > 1.0) of miR-93, miR-223 and miR-532, while in the remaining 41.5% the up-regulation trend was confirmed for one or two out of the three miRNAs.

**Fig. 2:**
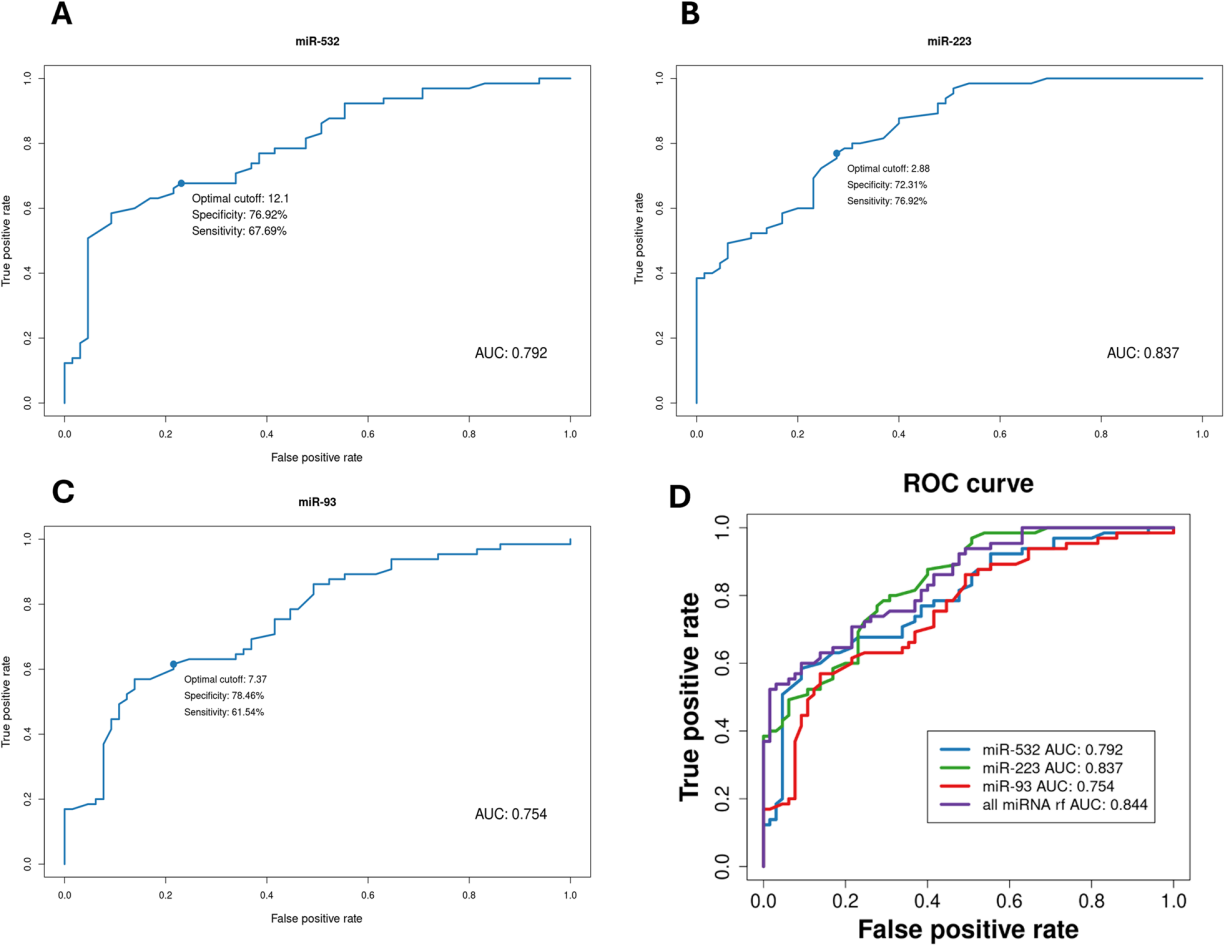
ROC analysis for serum miR-93, miR-223, and miR-532 and cumulative ROC curve. Receiver operating characteristic (ROC) curve analyses demonstrated the diagnostic ability of miR-93 (**A**), miR-223 (**B**) and miR-532(**C**) in LSCC. AUC, sensitivity and specificity was examined comparing deltaCT between LSCC (n = 65) and healthy cohort (n = 65). Cumulative ROC curve analysis (**D**) demonstrated the diagnostic ability of the simultaneous detection of miR-93 (**A**), miR-223 (**B**) and miR-532(**C**) in LSCC

To define the potential diagnostic value of serum miR-93, miR-223 and miR-532 levels, the receiver operating characteristic (ROC) curve was plotted. This technique served to identify a diagnostic threshold value for miRNA candidates. The area under the ROC curve (AUC) was 0.754 for miR-93, 0.837 for miR-223 and 0.792 for miR-532. The diagnostic sensitivity, specificity and CI values calculated for each one [miR-93 (Sensitivity = 61.54%, Specificity = 78.46%), miR-223 (Sensitivity = 76.92%, Specificity = 72.31%) and miR-532 (Sensitivity = 67.69%, Specificity = 76.92%)], showed their moderate diagnostic ability (Fig. 2A–C).

Moreover, we defined the potential diagnostic value of the miRNA signature (miR-93, miR-223 and miR-532), to build a diagnostic panel endowed with enhanced accuracy in distinguishing LSCC patients from healthy individuals, compared to each individual miRNA. To build the classifier the random forest model was used, while the validation method performed was the “leave-one-out". The resulting AUC was 0.844, thus increased with respect to the AUC values of the individual miRNAs. In addition, the diagnostic sensitivity and specificity respectively of 70.77% and 78.46% showed the potential diagnostic ability of the signature (Fig. 2D).

**Fig. 3:**
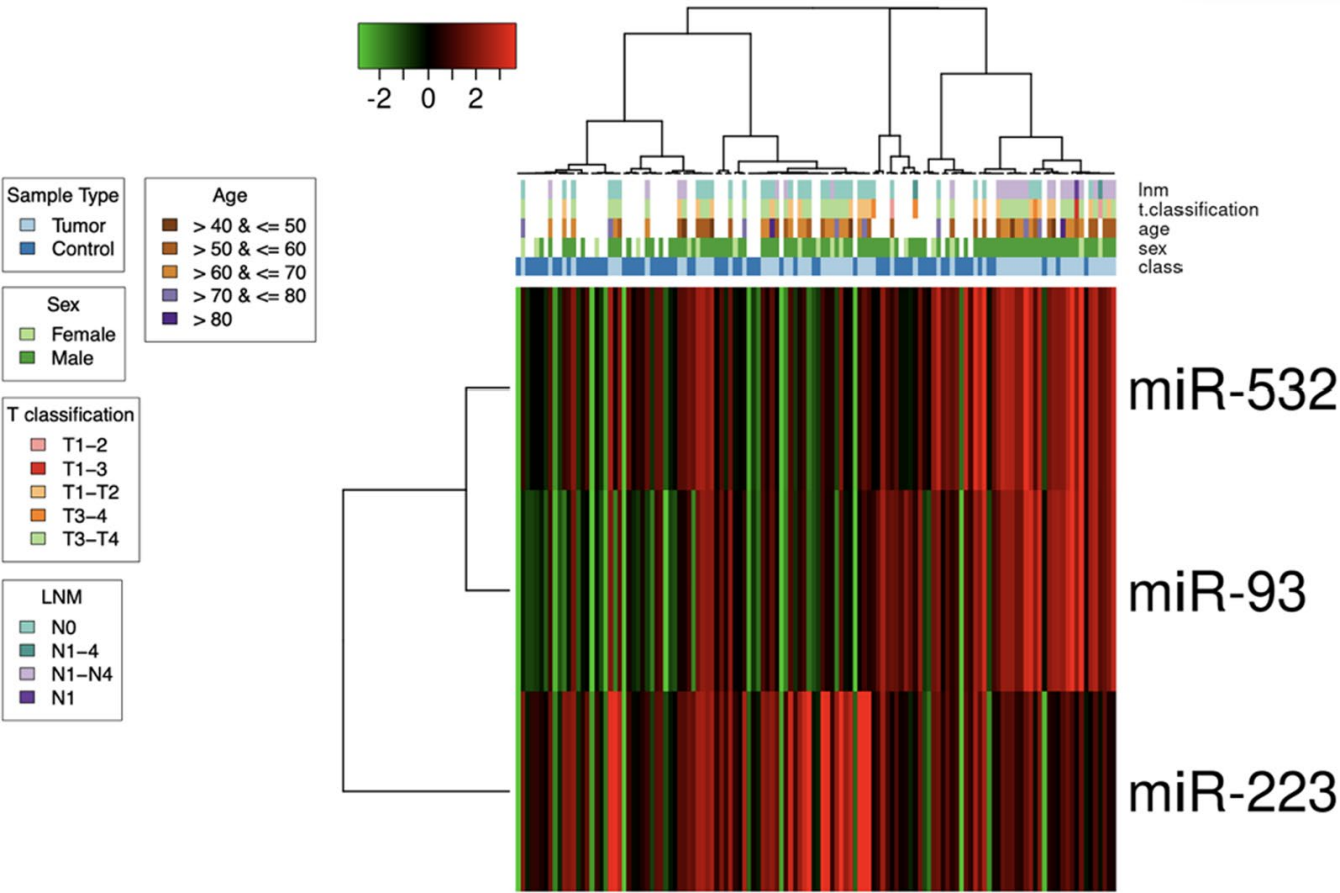
Heat map of the hierarchical clustering of the patient subgroups by the levels of miR-93, miR-223 and miR-532 for LSCC. The hierarchical clustering heatmap shows no significant correlation between miRNAs and the clinical information

In addition, to investigate the potential diagnostic power of the 3 miRNAs, we have evaluated their impact on prognosis. Therefore, based on our findings, we visualized a possible statistical relation between miRNA expression levels and LSCC patients’ clinical and pathologic features, by a clustering analysis. The analysis presented not a statistically significant distribution of miRNA expression in relation to age, gender, or lymph node metastases (Fig. 3).

Moreover, we have evaluated the impact on prognosis of the 3 miRNAs studying a possible statistical correlation between miRNA expression levels and clinicopathological features of the LSCC patients’ cohort **(**Table 4**)**. The Spearman’s correlation coefficient (*r*) and *p*-value was calculated for each of the 3 miRNAs. Interestingly, the results showed no significant correlation for the tumor stage and for the presence of lymph nodal metastases, suggesting a specific potential diagnostic value in detecting the LSCC in the analyzed categories.

**Table 4:** Correlation analysis of clinic-pathological parameters associated with miR-93, miR-223 and miR-532 level of expression in LSCC patients

Clinical Features	Patients (n)	MiR-93			MiR-223			Mir-532		
		Mean Log2 FC	r	p value	Mean Log2 FC	r	p value	Mean Log2 FC	r	p value
Age										
≤ 60	30	1,583,333	− 0,1695	0,0885	2,333,333	− 0,05514	0,3313	1,696,667	− 0,1943	0,0605
> 60	35	1,125,714			1,394,286			1,502,857		
Gender										
Male	56	1,380,357	− 0,08196	0,2582	1,8375	− 0,1615	0,0993	1,596,429	− 0,1248	0,1610
Female	9	1,066667			1,766,667			1,566,667		
Tumor stage										
T1-2	23	1,547,826	0,09438	0,2273	2,03913	− 0,02917	0,4088	1,817,391	0,006008	0,4811
T3-4	42	1,221,429			1,711,905			1,469,048		
LNM										
Negative	37	1,397,297	0,1127	0,1858	1,843,243	0,1384	0,1359	1,689,189	0,1276	0,1555
Positive	28	1,257,143			1,807,143			1,464,286		

### Bioinformatic analysis of MiRNA-related clinical data and predicted targets in cancer pathways

#### Kaplan–Meier survival analysis

**Fig. 4:**
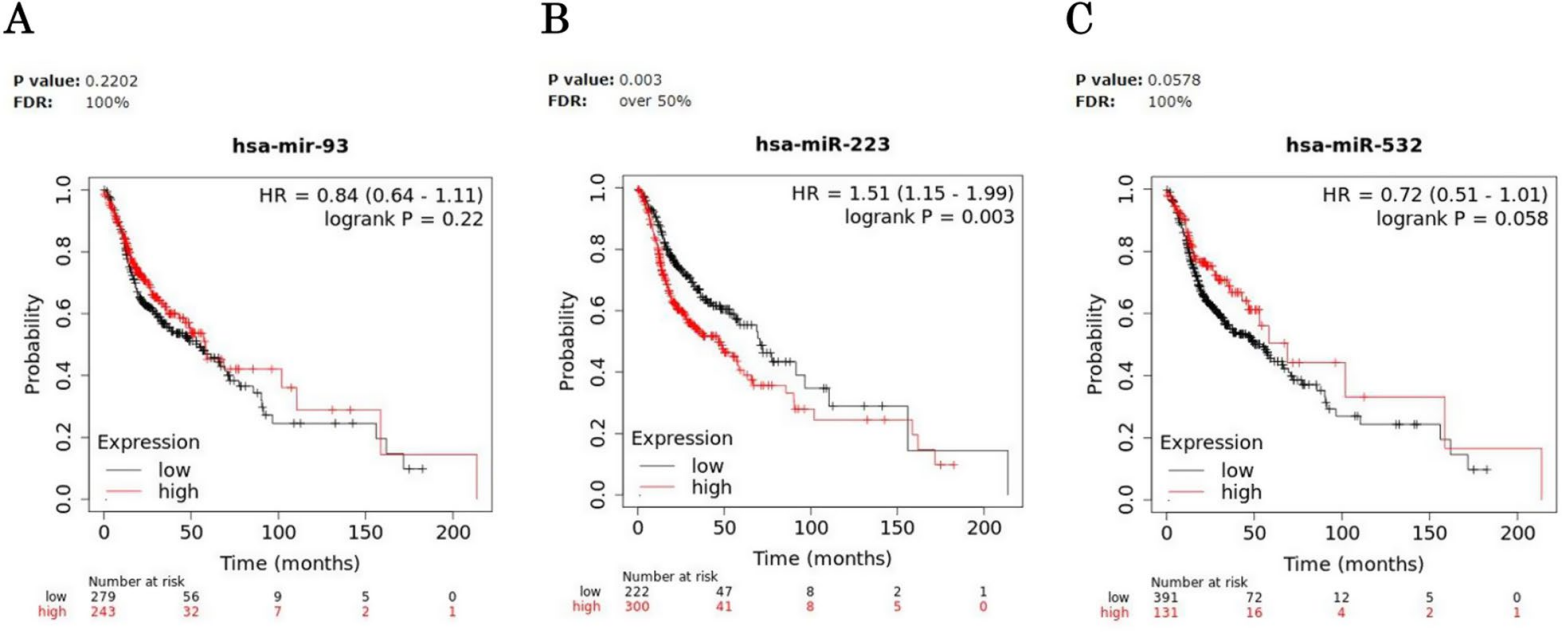
Survival analysis in HNSCC patients. Kaplan–Meier curves displaying the estimated survival probability for 2 different groups of HNSCC patients. **A** Patients either with low or high miR-93 expression levels, **B** patients either with low or high miR-223 expression levels, **C** patients either with low or high miR-532 expression levels. Hazard ratio (HR) and 95% confidence were calculated automatically by website tool. The values of each group are shown as the mean ± SD. p-value < 0.05 was regarded as statistically significant by using Log-rank test

We evaluated the prognostic significance of miR-93, miR-223 and miR-532 in Kaplan–Meier (KM) plotter online database (http://kmplot.com). In detail, we estimated the impact of miRNAs on survival in HNSCC; the correlation between the expression levels of each miRNA target and the overall survival (OS) rate was calculated by the Kaplan–Meier curve and log-rank test. Notably, the results of the Kaplan–Meier survival plots, showed that, among the three miRNAs, a meaningful association between high miR-223 expression levels and poor overall survival [HR = 1.51(1.15–1.99), logrank P = 0.003] was recorded in HNSCC (Fig. 4). Particularly, patients with high miR-223 expression had a median survival of 46.6 months, respect to patients with low levels, which showed a median survival of 70.67 months, after 240 months from disease onset (Fig. 4B). The log-rank test indicates a significant difference between the survival curves.

**Fig. 5:**
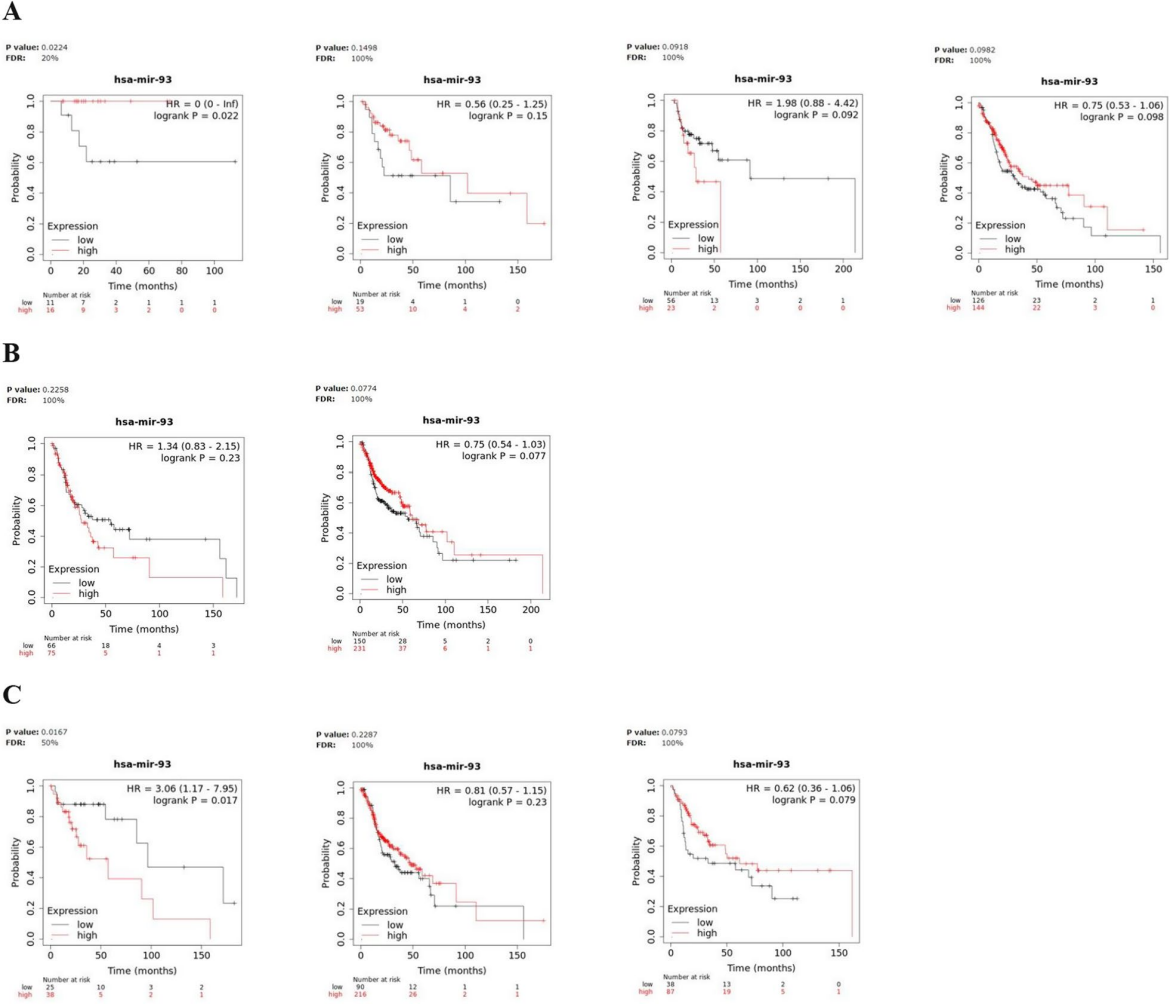
Survival analysis in miR-93 high- and low-expressing HNSCC groups. Kaplan–Meier curves displaying the estimated survival probability for 2 different groups of HNSCC patients in different physiological or clinical conditions, such as (**A**) tumor stage, **B** gender and (**C**) tumor grade. Hazard ratio (HR) and 95% confidence were calculated automatically by website tool. The values of each group are shown as the mean ± SD. p-value < 0.05 was regarded as statistically significant by using Log-rank test

**Fig. 6:**
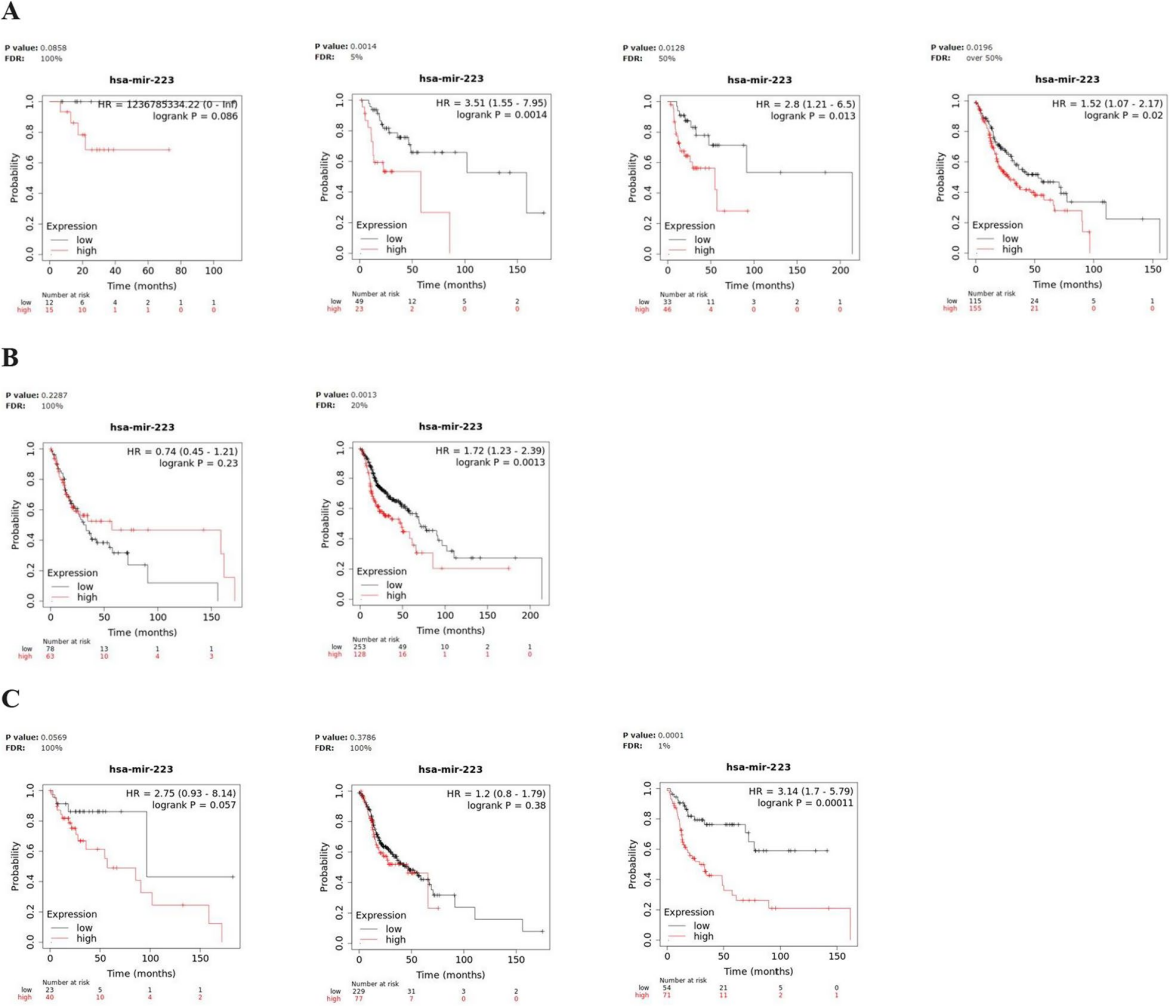
Survival analysis in miR-223 high- and low-expressing HNSCC groups. Kaplan–Meier curves displaying the estimated survival probability for 2 different groups of HNSCC patients in different physiological or clinical conditions, such as (**A**) tumor stage, **B** gender and (**C**) tumor grade. Hazard ratio (HR) and 95% confidence were calculated automatically by website tool. The values of each group are shown as the mean ± SD. p-value < 0.05 was regarded as statistically significant by using Log-rank test

**Fig. 7:**
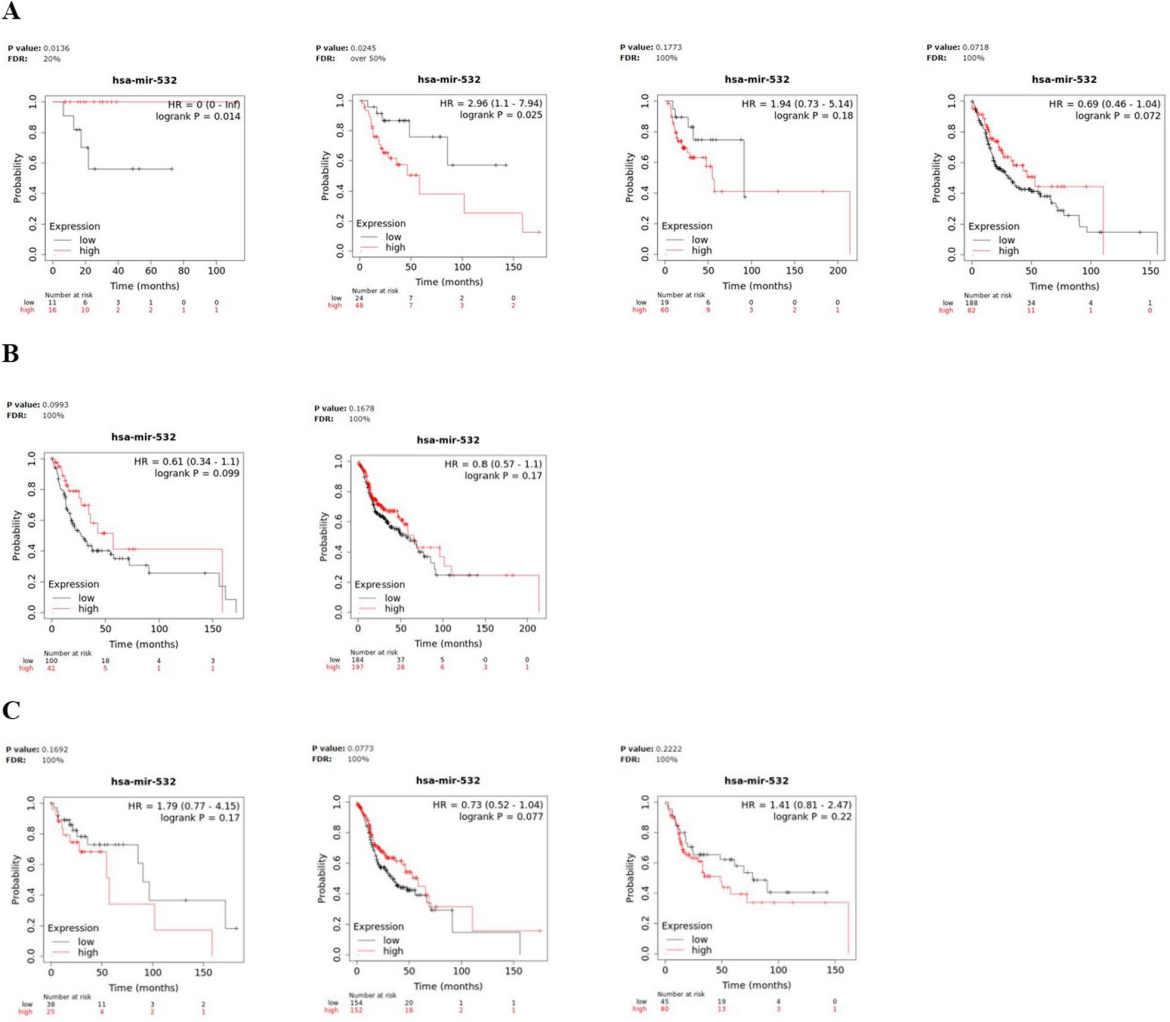
Survival analysis in miR-532 high- and low-expressing HNSCC groups. Kaplan–Meier curves displaying the estimated survival probability for 2 different groups of HNSCC patients in different physiological or clinical conditions, such as (**A**) tumor stage, **B** gender and (**C**) tumor grade. Hazard ratio (HR) and 95% confidence were calculated automatically by website tool. The values of each group are shown as the mean ± SD. p-value < 0.05 was regarded as statistically significant by using Log-rank test

Moreover, to further evaluate the prognostic significance of the miRNA panel in HNSCC, we investigated for the same patients, the relationship between miRNA expression and survival time in different physiological or clinical conditions, as gender and tumor stage or grade. Survival analysis for miR-93, miR-223 and miR-532 high- and low-expressing groups, based on clinical features was described in Figs. 5, 6 and 7 and the corresponding median survival rate (months) are resumed in Table 5.

**Table 5:** Median survival time in miR-93, miR-223 and miR-532 High- and low-expressing HNSCC patients in different physiological or clinical conditions

*Median survival (months)*									
	MiR-93			MiR-223			MiR-532		
	Low expression cohort	High expression cohort	HR and logrank P	Low expression cohort	High expression cohort	HR and logrank P	Low expression cohort	High expression cohort	HR and logrank P
Stage									
1	NA	NA		NA	NA		NA	NA	
2	85.67	101.97	0.56 (0.25–1.25) 0.15	158.67	58.27	3.51 (1.55–7.95) 0.0014	85.67	18.67	2.96 (1.1–7.94) 0.025
3	91.37	28.73	1.98 (0.88–4.42) 0.092	213.9	54.7	2.8 (1.21–6.5) 0.013	91.37	54.7	1.94 (0.73–5.14) 0.18
4	30.9	42.97	0.75 (0.53–1.06) 0.098	53.03	27.97	1.52 (1.07–2.17) 0.02	30.9	53.03	0.69 (0.46–1.04) 0.072
Gender									
Female	54.7	27.43	1.34 (0.83–2.15) 0.23	30.9	57.27	0.74 (0.45–1.21) 0.23	27.97	57.27	0.61 (0.34–1.1) 0.099
Male	55.7	61.27	0.75 (0.54–1.03) 0.077	70.67	48.87	1.72 (1.23–2.39) 0.0013	55.7	65.73	0.8 (0.57–1.1) 0.17
Grade									
*1*	96.67	57.27	3.06 (1.17–7.95) 0.017	96.67	57.27	2.75 (0.93–8.14) 0.057	90.57	57.27	1.79 (0.77–4.15) 0.17
*2*	32.67	47.67	0.81 (0.57–1.15) 0.23	46.6	46.47	1.2 (0.8–1.79) 0.38	32.83	58.73	0.73 (0.52–1.04) 0.077
*3*	33.27	61.27	0.62 (0.39–1.06) 0.079	69.43	11.37	3.14 (1.7–5.79) 0.000113	77.3	48.87	1.41 (0.81–2.47) 0.22

The analysis revealed that high-level miR-93 and lower tumor grade were potential risk factors significantly associated with poor OS in HNSCC patients [HR = 3.06 (1.17–7.95), logrank P = 0.017] (Fig. 5C).

On the other hand, high miR-223 levels negatively affected OS of HNSCC patients regardless of tumor stage (stage2: HR = 3.51 (1.55–7.95), logrank P = 0.0014), (stage3: HR = 2.8 (1.21–6.5), logrank P = 0.013), (stage4: HR = 1.52 (1.07–2.17), logrank P = 0.02) and in association with higher tumor grade (HR = 3.14 (1.7–5.79), logrank P = 0.00011) but predicted, at the same time, a more favourable OS for high-miR-223 expressing male HNSCC patients (HR = 1.72 (1.23–2.39), logrank P = 0.0013) (Fig. 6A–C).

Finally, for miR-532, high levels and lower tumor stage, were the risk factors that significantly contributed to worse OS in HNSCC patients (stage2: HR = 2.96 (1.1–7.94), logrank P = 0.025) **(**Fig. 7A**)**.

#### Exploring dysregulated miRNAs associated with tumor development, progression and clinical parameters in HNSCC

MiRNAs can act as either oncogenes or tumor suppressors affecting the hallmarks of cancer, including sustaining proliferative signaling, evading growth suppressors, resisting cell death, activating invasion and metastasis, and inducing angiogenesis. In this scenario dysregulated miRNAs represent a powerful diagnostic and prognostic tool especially if we consider their correlation with clinical parameters. The online resource OncomiR allowed us to explore miRNA dysregulation in HNSCC. We verified if miR-93, miR-223 and miR-532 were associated with tumor development or progression analysing correlations with tumor stage and grade, presence of lymph node and distant metastases or patients’ clinical features. MiR-93 and miR-223 expression were significantly associated to tumorigenesis for 16 cancer types, including HNSCC (Table 6). On the other hand, there are no data supporting a significant involvement of mir-532 in the HNSCC tumorigenesis.

**Table 6:** MiR-93 and miR-223 expression is significantly associated with tumorigenesis of HNSCC

miRNA name	Cancer type	T-test p-value	T-test FDR	Up-regulated in:	Tumor Log2 mean expression	Normal Log2 mean expression
miR-93	HNSCC	1.66e-11	3.58e-10	Tumor	12.26	10.93
miR-223	HNSCC	1.30e-02	2.83e-02	Tumor	9.13	8.44

In addition**,** all three miRNAs showed significant correlations with some clinical HNSCC patients’ characteristics, and, among them, all miRNAs were associated to tumor histological grade. MiR-93 expression significantly correlated with clinical M and N status (p-value 1.32e-03 and 1.37e-02 respectively), histologic grade (p-value 2.78e-02), pathologic M status (p-value 1.95e-03) and sex (p-value 1.44e-02) **(**Table 7**)**. The greatest impact on cancer-related processes was observed for miR-223 whose expression resulted significantly correlated with clinical M, N and T status (p-value 7.10e-01, 5.54e-01 and 9.04e-01 respectively), clinical and pathologic stage (p-value 2.53e-01 and 5.08e-02 respectively), histological grade (p-value 1.58e-02), pathologic N and T status (p-value 7.47e-02 and 3.84e-01 respectively) and sex (p-value 3.86e-04) (Table 7). On the other hand, miR-532 showed significant correlations limited to clinical T status (p-value 1.61e-03), histological grade (p-value 9.52e-07) and pathologic T status (p-value 3.03e-03) (Table 7).

**Table 7:** Clinical parameters associated with miR-93, miR-223 and miR-532 in HNSCC

Clinical parameter	miRNA correlated	ANOVA p-value	ANOVA FDR	Multivariate log rank p-value	Multivariate log rank FDR
Clinical M status	miR-93	1.32e−03	1.77e−02	7.11e−01	9.69e−01
	miR-223	7.10e−01	9.80e−01	8.35e−03	6.90e−02
Clinical N status	miR-93	1.37e−02	1.62e−01	5.55e−01	8.58e−01
	miR-223	5.54e−01	8.80e−01	4.48e−03	4.01e−02
Clinical T status	miR-223	9.64e−01	9.86e−01	8.62e-03	7.05e−02
	miR-532	1.61e−03	4.59e−02	3.86e−01	7.32e−01
Clinical stage	miR-223	2.53e−01	7.44e−01	5.56e−03	4.98e−02
Histological grade	miR-93	2.78e−02	8.20e−02	5.80e-01	8.86e−01
	miR-223	1.58e−02	5.05e−02	1.21e−02	8.41e−02
	miR-532	9.52e−07	1.30e−05	2.67e−01	5.93e−01
Pathologic M status	miR-93	1.95e−03	5.00e−02	4.12e−01	7.38e−01
Pathologic N status	miR-223	7.47e−02	3.08e−01	2.35e−03	4.25e−02
Pathologic T status	miR-223	3.84e−01	7.28e−01	1.61e−02	1.15e−01
	miR-532	3.03e−03	4.70e−02	5.62e−02	8.86e−01
Pathologic stage	miR-223	5.08e−02	5.68e−01	1.14e−02	8.74e−02
Sex	miR-93	1.44e−02	2.16e−01	8.72e−01	9.97e−01
	miR-223	3.86e−04	2.33e−02	2.37e−02	1.44e−01

#### *In-silico* analysis of common candidate targets

**Fig. 8:**
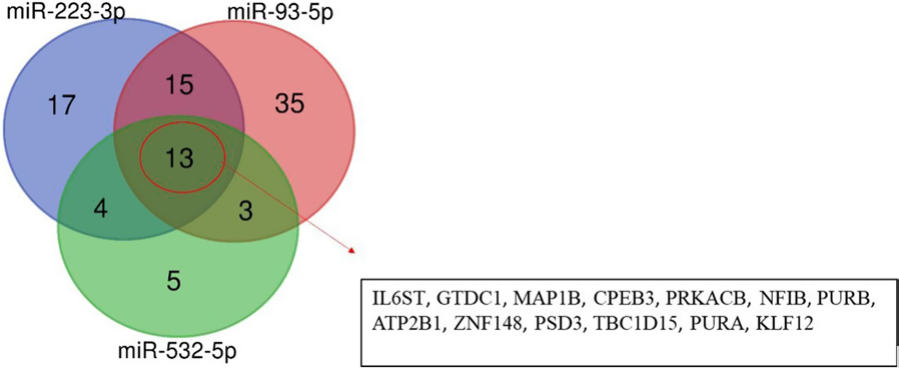
Venn Diagram of the molecular targets cross-regulated by miR-93, miR-223 and miR-532. The Venn plot highlights 13 common targets between miR-93, miR-223 and miR-532, 15 between miR-93 and miR-223, 3 for miR-93 and miR-532 and 4 for miR-223 and miR-532, respectively. The 13 genes shared by all three miRNAs are zoomed

The increased expression levels of the three-miRNA panel recognized in LSCC serum samples, as well as the outcome of the bioinformatic analyses suggesting a prognostic potential, supported the hypothesis that such miRNAs could play a pro-tumorigenic role. Thus, to better clarify the molecular bases of this function, we focused on the possible cross-modulation of the putative tumor-suppressor targets. For this purpose, we queried miRTargetLink 2.0 which provides a large set of miRNA gene associations from different in silico platforms [miRTarBase, mirDIP, miRDB and miRATBase] and allows to detect putative pathways through which miRNAs could exert their biological role. In details, we screened, for each up regulated miRNA, the gene targets predicted from at least three bioinformatic databases. A total of 49, 66 and 25 targets were selected for miR-223, miR-93 and miR-532, respectively, and 13 overlapping genes (IL6ST, GTDC1, MAP1B, CPEB3, PRKACB, NFIB, PURB, ATP2B1, ZNF148, PSD3, TBC1D15, PURA, KLF12) were identified using a Venn diagram, as genes commonly regulated by miR-93, miR-223 and miR-532 simultaneously (Fig. 8).

#### Enrichment analysis of co-regulated genes and network construction

**Fig. 9:**
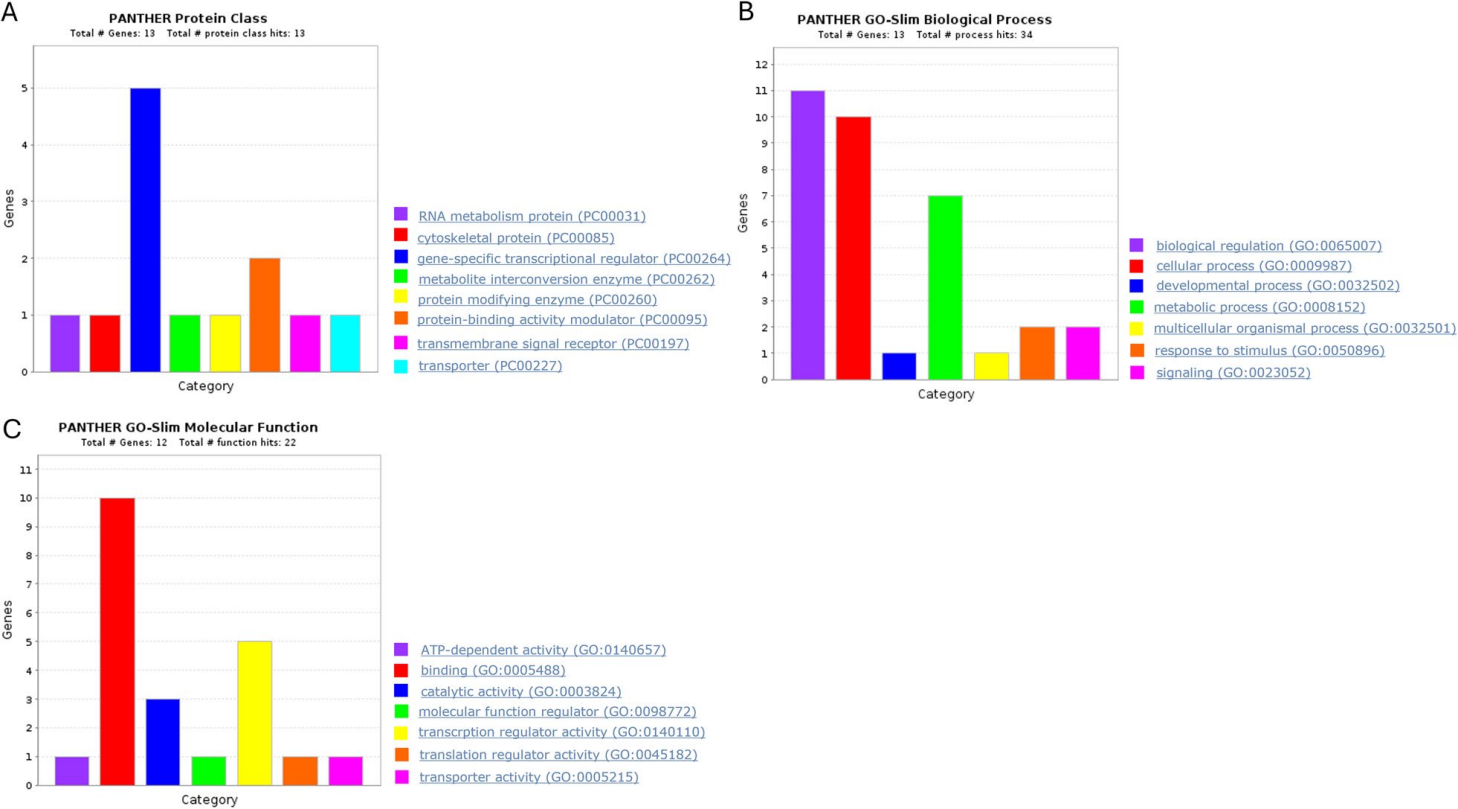
In silico analysis of common candidate targets performed by PANTHER software (v16.0). **A** Molecular common target classification. The bar graph depicts the stratification of the 13 co-regulated gene targets in protein classes. 5 genes are classified as gene-specific trascriptional regulators, while 2 belong to the protein-binding activity modulators. Each of the remaining 6 genes represents a single category. **B** Involvement of the common molecular targets in biological processes. The bar graph depicts the pathways involving the 13 gene targets shared by miR-93, miR-223 and miR-532. The “biological regulation”, “cellular process” and “metabolic process”are the top 3 groups of biological processes shared by the 13 co-regulated genes. **C** Main molecular functions of the cross-regulated gene targets. The bar graph depicts the molecular functions of the 13 gene targets shared by miR-93, miR-223 and miR-532. 10 genes share the binding molecular function and 5 genes share the transcription regulator activity

To investigate the biological function of the 13 predicted cross-regulated targets, protein classification and pathway enrichment analyses were performed using PANTHER software; this is able to visualize the relationship between genes and specific signaling pathways in which they are involved (Fig. 9A–C). As shown in Fig. 9A, we found that the 13 common targets can be classified in 8 protein classes and, in details, these are proteins belonging to the classes of “gene-specific transcriptional regulators” (NFIB, PURB, ZNF148, PURA, KLF12) and “protein-binding activity modulators” (PSD3, TBC1D15).

The analysis also revealed that 13 common gene targets were significantly enriched in 7 different biological processes **(**Fig. 9B**)**. Among them, “biological regulation” (IL6ST, MAP1B, PRKACB, NFIB, PURB, ATP2B1, ZNF148, TBC1D15, PURA, KLF12, CPEB3), including tumor promoting inflammation members, and “metabolic process” (CPEB3, PRKACB, NFIB, PURB, ZNF148, PURA, KLF12) caught our attention.

In terms of molecular functions, the common gene targets were mainly associated with “binding” (MAP1B, CPEB3, PRKACB, NFIB, PURB, ATP2B1, ZNF148, TBC1D15, PURA, KLF12), “catalytic activity” (PRKACB, ATP2B1, TBC1D15) and “transcription regulator activity” (NFIB, PURB, ZNF148, PURA, KLF12) (Fig. 9C).

**Fig. 10:**
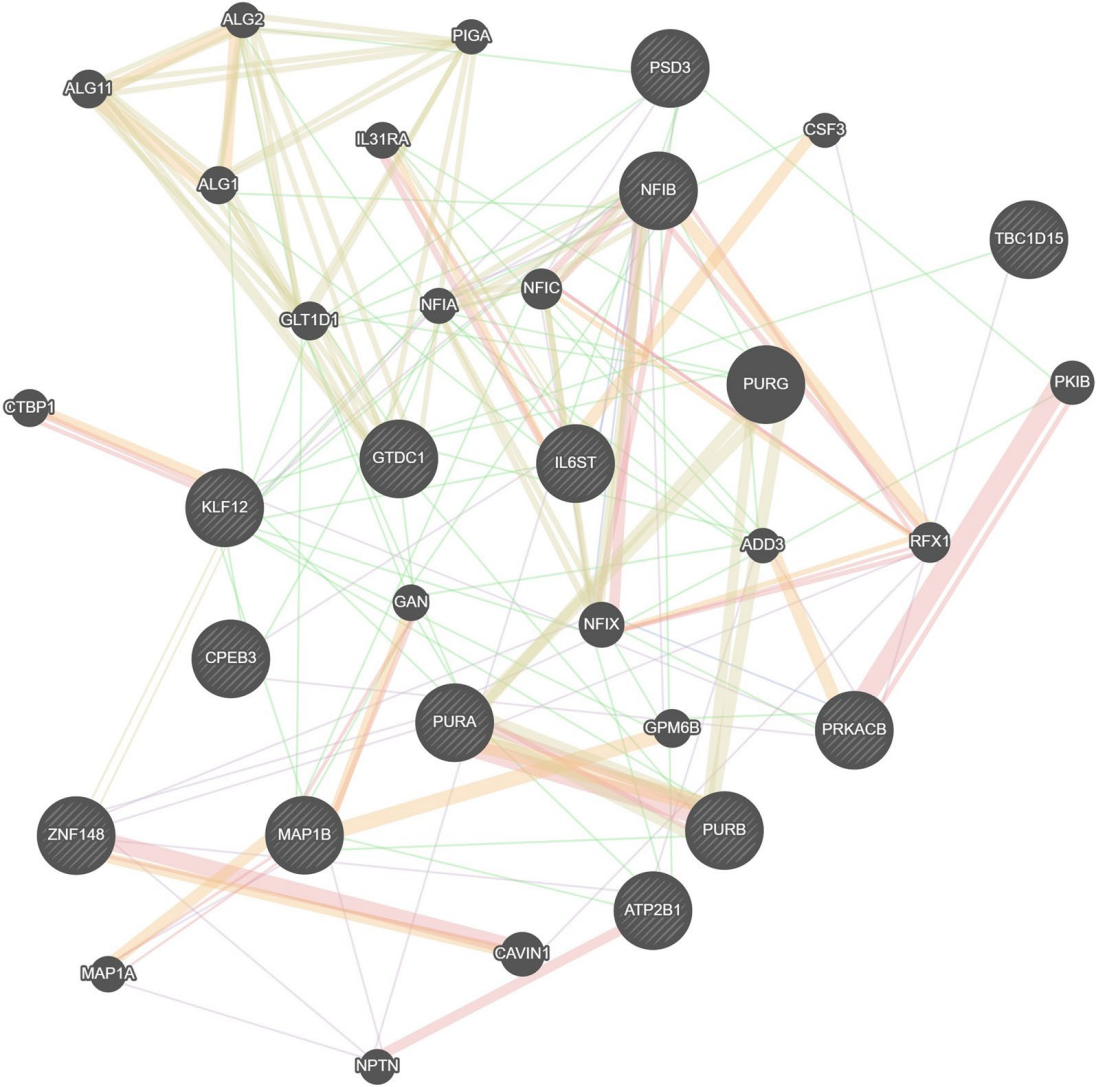
Protein interactions among gene targets. Network image showed 33 nodes (genes): 13 gene targets shared by miR-93, miR-223 and miR-532 and 20 related genes, and underlined possible co-expressions, co-localizations, physical interactions, shared protein domains and functions among them

Protein interactions among gene targets were predicted by the GeneMANIA online platform. This provides an algorithm that calculates network relevance to predict genes likely to share the functions among the query list, by assigning to each a weight. The analysis revealed 34.58% of physical interactions between target and related genes and predicted the co-expression between ZNF148 and PURA or NFIB, KLF12 and PSD, among the most important. Particularly, ZNF148 and KLF12 shared protein domain, while IL6ST, PRKACB, NFIB were often co-localized. Genes’ interaction and its possible correlation with a specific function, was studied with this software and the main mechanism that involves 3 out of the 33 genes with a statistical significance is represented by the mannosyltransferase activity (Fig. 10).

As part of the bioinformatic analysis aimed at deepening the role of miRNA signature regulating genes in cancer related pathways, we also explored Reactome Software. 6 out of 13 identifiers were found 109 pathways were hit by at least one of them. In detail, Table 8 summerizes the 25 most relevant pathways sorted by p-value.

**Table 8:** The 25 most relevant pathways sorted by p-value

Pathway name	Entities				Reactions	
	Found	Ratio	*p*-value	FDR*	Found	Ratio
Interleukin-6 signaling	3/15	0.001	9.29e−07	1.05e−04	15/20	0.001
Interleukin-6 family signaling	3/28	0.002	5.99e−06	3.35e−04	19/34	0.002
PKA-mediated phosphorylation of key metabolic factors	1/5	4.25e−04	0.006	0.095	3/5	3.36e−04
HDL assembly	1/8	6.79e−04	0.01	0.095	1/9	6.05e−04
ROBO receptors bind AKAP5	1/9	7.64e−04	0.011	0.095	1/7	4.70e−04
MAPK1 (ERK2) activation	1/10	8.49e−04	0.013	0.095	1/3	2.02e−04
Interleukin-27 signaling	1/11	9.34e−04	0.014	0.095	8/20	0.001
MAPK3 (ERK1) activation	1/11	9.34e−04	0.014	0.095	1/4	2.69e−04
CREB1 phosphorylation through the activation of Adenylate Cyclase	1/12	0.001	0.015	0.095	4/6	4.03e−04
Interleukin-35 Signalling	1/12	0.001	0.015	0.095	10/26	0.002
Reduction of cytosolic Ca + + levels	1/12	0.001	0.015	0.095	1/3	2.02e−04
Regulation of glycolysis by fructose 2,6-bisphosphate metabolism	1/12	0.001	0.015	0.095	1/4	2.69e−04
Signaling by Interleukins	3/470	0.04	0.02	0.095	36/505	0.034
Rap1 signalling	1/16	0.001	0.02	0.095	1/7	4.70e−04
IL-6-type cytokine receptor ligand interactions	1/17	0.001	0.021	0.095	4/14	9.40e−04
PKA activation	1/18	0.002	0.023	0.095	2/4	2.69e−04
PKA activation in glucagon signalling	1/18	0.002	0.023	0.095	1/2	1.34e−04
Plasma lipoprotein assembly	1/19	0.002	0.024	0.095	1/19	0.001
PKA-mediated phosphorylation of CREB	1/20	0.002	0.025	0.095	4/7	4.70e−04
CD209 (DC-SIGN) signaling	1/22	0.002	0.028	0.095	1/11	7.39e-04
RNA Polymerase III Transcription Termination	1/23	0.002	0.029	0.095	1/2	1.34e−04
DARPP-32 events	1/24	0.002	0.03	0.095	4/12	8.06e−04
RAF-independent MAPK1/3 activation	1/24	0.002	0.03	0.095	2/12	8.06e−04
Triglyceride catabolism	1/24	0.002	0.03	0.095	2/17	0.001
Platelet calcium homeostasis	1/28	0.002	0.035	0.095	1/8	5.37e−04

^*^False discovery rate

**Fig. 11:**
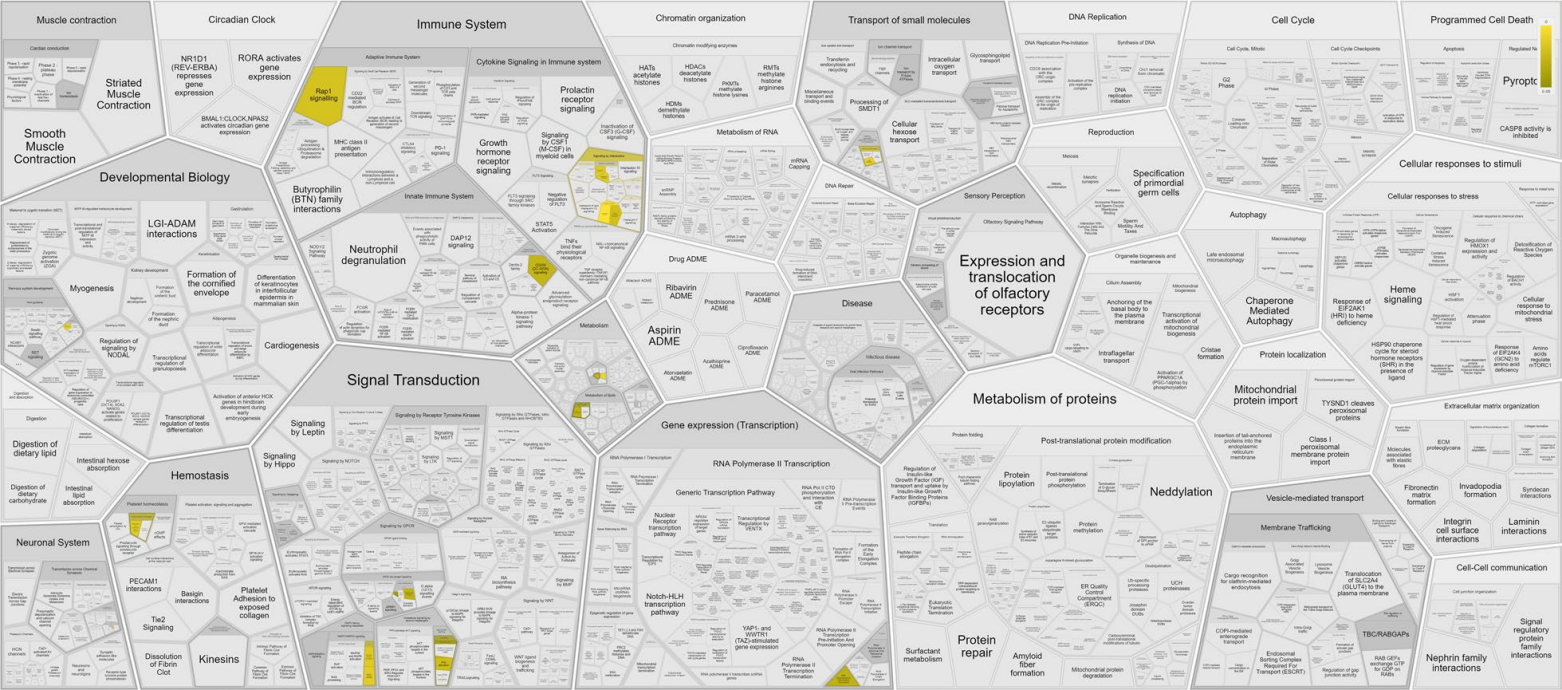
Genome-wide overview of the pathway analysis. Reactome pathways are arranged in a hierarchy. The center of each of the circular “bursts” is the root of one toplevel pathway. Each step away from the center represents the next level lower in the pathway hierarchy. The color code denotes over-representation of that pathway; light grey signifies pathways which are not significantly over-represented

Most of these pathways, involve two of the commonly regulated genes, IL6ST and PRKACB, that actively participate in several processes including cell proliferation, differentiation, immunity and metabolism. IL6ST acts through homodimer formation with dimeric complexes between IL6 or IL11 and their corresponding receptors, IL6R and IL11R. The resulting hexameric or higher order complex can thus induce anabolic signal initiation. Regarding PRKACB protein, the major role consists in the stimulation of cell proliferation and differentiation through cAMP (Fig. 11**)**.

### Preliminary in vitro results

Among the differentially expressed miRNAs, miR-223 showed the highest median fold change (3.3, p-value = 1.8 × 10^–12^) and the most promising diagnostic potential (AUC = 0.837). Therefore, we profiled its basal expression in a panel of three HNSCC cell lines (HEp-2, HNO-210, FaDu) compared to a healthy spontaneously immortalized human keratinocyte cell line (HaCat) (Figure S1 A) by qRT-PCR. High endogenous levels of miR-223 were assessed for HNO-210 cell lines, while a low basal miR-223 expression was underlined for HEp-2 and FaDu cell lines. HNO-210 were then selected to construct an in vitro model to preliminary investigate miR-223 biological functions. We performed both gain- and loss-of-function study through transient transfection by Lipofectamine 2000 with miR-223 Mimic and miR-223 Inhibitor, besides their negative controls (NC Mimic- NC Inhibitor), represented by the respective scramble sequences. Then, we determined the transfection efficiency by qRT-PCR (Figure S1 B). As shown in Figure S1 B, a conspicuous up-regulation of miR-223 expression was induced by miR-223 Mimic compared to NC Mimic (p < 0.001) and a slight down regulation was observed upon Inhibitor transfection compared to NC Inhibitor. MTT (3-(4,5- dimethylthiazol-2-yl)-2,5-dimethyltetrazolium bromide) cell viability assay showed a significant increase in the proliferation ability of miR-223 Mimic compared to NC Mimic transfected cells at 48–72 h (p < 0.0) (Figure S2), while a mild suppression of proliferation was observed for miR-223 Inhibitor compared to NC transfected cells at 72 h (p < 0.05). These results suggest miR-223 overexpression as a tumor promoting function which deserves further investigation in forthcoming studies.

## Discussion

Laryngeal cancer is one of the most common malignancy of head and neck. During the past three decades, its incidence and prevalence have increased by 12.0% and 23.8% respectively, with more than 180,000 new cases reported yearly worldwide, ranking as the 22nd in incidence and the 18th in prevalence and mortality [31]. The most common histological type is laryngeal squamous cell carcinoma (LSCC), accounting for over 90% of the malignancy [1].

Among all the treatments, the options for advanced LSCC stages usually include a combination of chemotherapy and radiotherapy, while surgical resection and radiation therapy are currently used for early-stage cancers. Compared to the past, LSCC surgery is less invasive, tending to organ preservation and used only for rescue treatment or for injuries with extra-laryngeal extension or cartilage destruction. However, due to the absence of specific symptoms and reliable markers, LSCC is usually diagnosed in advanced stages, resulting in delayed treatment, high frequency of tumor recurrence, metastasis and worse prognosis.

miRNA identification into the bloodstream or other biological fluids, has generated great interest for the potential use as biomarkers. Circulating miRNAs, are a new class of gene regulators, whose role in cancer onset and progression has been deepened, opening new opportunities for therapeutic application. They belong to the family of small non-coding RNAs, molecules of ~ 24 nt, that inhibit mRNA translation and/or negatively regulate its stability. In the last years, an increasing number of dysregulated miRNAs in plasma or serum, have been considered as novel hallmarks of cancer.

A large body of evidence showed aberrant miRNA regulation in LSCC tissues and/or plasma [32–35], pointing out a strong correlation between their expression levels and the main processes underlying tumor initiation and progression, such as proliferation, migration, invasion, metastasis, tumor infiltration, and disease relapse. Based on the modulation trend and on bioinformatic analysis of the putative targets, several reports have highlighted a possible oncogenic or tumor-suppressive role for several miRNAs in LSCC pathogenesis, although further insights are still required for their ultimate use as diagnostic, prognostic and therapeutic tools.

In this scenario, the objective of the present manuscript is to determine a miRNA signature for the definition of LSCC diagnosis and prognosis, as well as to analyse the putative functional role of the identified miRNAs.

First of all, a high-throughput PCR array analysis on 45 patients, including 22 with lymph node metastases (N^+^) and 23 without (N^─^), was performed and compared to 23 healthy volunteers. MiRNA profiling allowed the detection of 81 dysregulated miRNAs (Fig. 1A), among which 11 significantly up-regulated—miR-532, miR-93, miR-451, miR-140, miR-223, miR-16, miR-20b, miR-29a, miR-132, miR-25, miR-20a—and 5 significantly down-regulated—miR-95, miR-150, miR-891a, miR-331 and miR-374 (Table 2). Based on the microarray analysis and on the additional validation by qRT-PCR in a small cohort of samples (data not showed), we focused on miR-532, miR-93 and miR-223 as up-modulated candidates. Consistently with the microarray results, we confirmed their significant up-regulation on an increasingly large cohort of LSCC patients (65 patients, divided into 28 N^+^ and 37 N^─^) (Fig. 1B). The selected miRNAs were also investigated for their diagnostic value both individually (Fig. 2A-C) and taken together (Fig. 2D), using ROC curve analysis. As a result, a moderate diagnostic potential was revealed for each one, and an increased AUC value, together with a globally improved sensitivity, was observed when we considered the diagnostic power of the combined signature. This outcome is certainly affected by the low sample size; therefore, the progressive expansion of study population will be indispensable to make a definitive elucidation. In the cohort herein analyzed, no correlation was detected between miRNA levels and clinicopathologic features like sex, age, lymph node metastasis involvement and T stage (Fig. 3).

miR-93, miR-223 and miR-532 are involved in carcinogenesis, progression and metastasizing of different neoplasms and many studies dissected their role in head and neck squamous cell carcinoma (HNCCS).

miR-93 is overexpressed in LSCC tissues [33] and it works as an oncogene in LSCC cells increasing proliferation, migration and invasion and inhibiting apoptosis [30]. High miR-93 expression has been also observed in HNSCC tissues and is correlated to cancer progression, metastasis and poor prognosis [31]. As well, a high-throughput qRT-PCR array for miRNA profiling in LSCC patients, showed a significant up-regulation of plasma miR-93 levels in cancer group compared to control ones [34]. Regarding miR-223, its up-regulation in oral squamous cell carcinoma (OSCC) tissues and cell lines increased cell growth and migration and suppressed apoptosis through direct targeting tumor suppressor FBXW7 [38]. In HNSCC tissues, it has been reported a significant correlation between miR-223 high expression and neutrophil infiltration. Moreover, in HNSCC cells, miR-223 ectopic expression increased proliferation, apoptosis and resistance to Cetuximab, also inducing pERK2, pAKT and AKT expression and angiogenesis inhibition [39]. Furthermore up-regulated levels of miR-223 were found in laryngeal neuroendocrine carcinomas, suggesting its oncogenic role not only in SCC but also in other subtypes [40]. Cancer-related miR-532 function has been characterized in several neoplasms excepting LSCC, hence additional evaluation of both expression levels and biological functions could be remarkable.

Based on these findings, the high expression levels of miR-93, miR-223 and miR-532 detected in LSCC represent an important starting point to better clarify their role in carcinogenesis and their powerful potential for LSCC minimally invasive diagnosis and/or prognosis.

To better understand the oncogenic effect and the prognostic significance of miR-93, miR-223 and miR-532, thorough bioinformatic analysis was performed. Firstly, by KM plotter online database, we estimated the effect of miRNAs on HNSCC patients’ survival and, notably, the results showed that high miR-223 levels associated to poor overall survival (Fig. 4B). Moreover, for the same patients, high miR-93 levels correlated with lower tumor grade (Fig. 5C), and high miR-532 levels with lower tumor stage (Fig. 7A) significantly contributing to worse OS. Moreover, increased miR-223 expression correlated to both high tumor stage and grade and negatively affected OS of HNSCC patients (Fig. 6A–C). This in silico analysis, performed on 523 HNSCC patients, revealed prominent prognostic information that our cohort of 65 patients lacked to show. Besides the sample size, another difference between our cohort and the patients screened by the queried bioinformatic tool is the inclusion, in the latter group, of multiple neoplasms of the head-neck district. To analyse the molecular basis underlying the pro-tumorigenic role of miR-93, miR-223 and miR-532, we focused on the common modulation of their main gene targets. We queried in silico miRNA target prediction tool (miRTargetLink 2.0) obtaining a total of 49, 66 and 25 gene targets for miR-223, miR-93 and miR-532, respectively. The derived Venn diagram identified 13 overlapping genes (IL6ST, GTDC1, MAP1B, CPEB3, PRKACB, NFIB, PURB, ATP2B1, ZNF148, PSD3, TBC1D15, PURA, KLF12), commonly regulated by miR-93, miR-223 and miR-532 simultaneously (Fig. 8). The list of genes commonly regulated by all three miRNAs, was analyzed with PANTHER and GeneMANIA software for miRNA pathway enrichment analysis. The analysis showed the involvement of the 13 common targets in 7 different biological processes, among which biological regulation (IL6ST, MAP1B, PRKACB, NFIB, PURB, ATP2B1, ZNF148, TBC1D15, PURA, KLF12, CPEB3) including tumor promoting inflammation, and metabolic process (CPEB3, PRKACB, NFIB, PURB, ZNF148, PURA, KLF12) were the most interestingly associated to tumor phenotype (Fig. 9B). The most significant genes included IL6ST, PRKACB, NFIB, ATP2B1, ZNF148 and CPEB3, all playing well known roles in different pro-tumorigenic processes occurring in solid tumors. In particular, IL6ST and PRKACB are involved in the majority of these signaling pathways mainly including cell proliferaton, differentiation, immunity and metabolism (Fig. 11).

IL6ST is a signal transducer through which cytokines, such as IL6, perform pleiotropic functions in many cancer-related processes including inflammatory pathways. In breast cancer, several studies highlighted IL6ST as a positive prognostic factor. In fact, it was downregulated in triple negative breast cancer, with higher expression correlated to better prognosis [41].

PRKACB has a central role in cAMP/PKA-induced signal transduction, a mechanism involved in cell proliferation, apoptosis, gene transcription, metabolism and differentiation. In non-small cell lung cancer (NSCLC) the effect of PRKACB upregulation was studied both in vitro and ex vivo. The findings suggested that NSCLC tissues showed much lower levels of both PRKACB mRNA and protein than the corresponding normal tissues and in transfected cancer cell lines, PRKACB upregulation reduced the number of proliferative, clonogenic, and invasive cells, while apoptosis rates was increased[42].

NFIB is a transcription factor responsible of the correct development and regulation of cell differentiation in different tissues even if recent evidence suggested its key role in cancer. In fact, it is a metastatic factor in small cell lung cancer and melanoma, but it also exhibits tumor suppressor functions in many other neoplasms. The contradictory effect of NFIB on tumor development and progression depends on the cellular and tissue molecular context [43].

ATP2B1is a plasma membrane Ca^2+^ ATPase important for intra-cellular calcium regulation and for calcium homeostasis and blood pressure control. It has been recently investigated the correlation between the ATP2B1 gene and the immune microenvironment in intrahepatic cholangiocarcinoma (ICC). As a result, ATP2B1 was identified as a prognostic factor, since its expression was positively correlated with immune scores, levels of infiltrating CD8 +and CD4 +T cells. Immunohistochemistry confirmed that the amount of CD8 +and CD4 +T cells was significantly higher in ICC tissue samples compared to ATP2B1-overexpressing tissues [44].

ZNF148 is a transcription factor whose downregulation seems to be a recurring event in different human cancers. These data suggest a putative tumor suppressor function for ZNF148 by regulating cell proliferation and differentiation. Its expression increases in the normal mucosa of colorectal cancer and remains high in tumor tissue at earliest stages, decreasing at advanced ones, thus suggesting an inverse correlation with malignant phenotypes. Moreover, ZNF148 act as a prognostic factor as its low expression is significantly associated to lymph node metastasis, poor differentiation, higher rate of disease recurrence, worse overall survival (OS) and shorter disease-free survival [45].

In colorectal cancer (CRC), CPEB3 is involved in the crosstalk between cancer cells and TAMs by targeting IL-6R/STAT3 signalling. TAMs enhanced both proliferation and invasion of CRC cells via IL-6, and then activated the IL-6R/STAT3 pathway. However, CPEB3 reduced IL-6R protein levels by directly binding IL-6R mRNA, leading to both increased expression of phosphorylated STAT3 in CRC cells and inhibition of epithelial-mesenchymal transition [46].

Collectively, our findings suggest that these three miRNAs identified in LSCC serum samples could collectively contribute to the targeting and the regulation of multiple factors involved in pro-tumorigenic pathways. Moreover, we studied in vitro the effect on viability of one of the three selected miRNAs, miR-223, which showed the highest median fold change (3.3, p-value = 1.8 × 10–12), and the most promising diagnostic potential (AUC = 0.837). Within the LSCC cell line panel screened for miR-223 basal levels, we chose to transfect HNO-210 cells that showed a basal over-expression of miR-223 (Figure S1A). The MTT assay underlined a significant increase of cell viability at 48 h and 72 h after transfection with miR-223 Mimic and an opposite effect at 72 h from transfection with miR-223 Inhibitor, both compared with the corresponding negative controls (Figure S2). This result confirmed miR-223 involvement in pro-tumorigenic processes.

## Strengths and weaknesses

One of the strengths of our study has been the identification of a three-miRNA molecular signature which increases the accuracy of detection and improves the specificity and sensitivity of diagnostic potential, compared to each individual miRNA. In addition, serum biomarkers have the undeniable advantage of being easily determined by quick and non-invasive procedures. Of note, the signature herein described has been tested and validated on a large cohort of 65 LSCC patients. An apparent limitation of the study could be found in the overlapping of 45 LSCC patient from the discovery cohort with the final number of 65 LSCC patients tested. Nevertheless, an independent analysis conducted on the 20 LSCC patients specifically enrolled for the validation set confirmed the significant upregulation trend previously found (Figure S3). Another critical point emerged during our study regards the correlation between miRNA expression levels and patients’ clinical-pathological data found by bioinformatic analysis, but not confirmed whitin our cohort of patients. In this regard, we have already mentioned the different sample size between the patients enrolled for the study and those analyzed by the publically available databases, as well as the inclusion in the in silico analysis of patients affected by neoplasms encompassing all the districts of the head-neck region.

## Conclusions

In conclusion, we have identified, for LSCC cancer patients, a possible miRNA signature for early diagnosis of disease. Moreover, analysing the putative targets shared by miR-93, miR-223 and miR-532, we assumed that together they could orchestrate the regulation of multiple cancer-related processes, such as inflammation, angiogenesis, apoptosis, proliferation, migration, and invasion, These findings, together with the current evidence in literature, provide the rationale for additional experiments aimed at better understanding the molecular bases of the cumulative oncogenic function played by all three miRNAs, through the characterization of their tissue expression profile, as well as the identification of the main genes that take part in miRNA-related cellular processes for the additional development of novel therapeutic opportunities based on the use of short single-stranded oligonucleotides acting as miRNA antagonists in cancer.

### Supplementary Information

Additional file 1.

## Data Availability

Datasets used and/or analyzed during the current study are available from the corresponding author on reasonable request.

## Abbreviations

LSCC: Laryngeal Squamous Cell Cancer
HPV: Human Papillomavirus
EMT: Epithelial–mesenchymal transition
miRNA: MicroRNA
AGO: Argonaute protein family
HNSCC: Head and Neck Squamous Cell Cancer
ROC: Receiver Operating Characteristics
AUC: Area Under Curve
N^─^: Lymph Node Metastasis Negative
N^+^: Lymph Node Metastasis Positive
(r): Spearman’s correlation coefficient
OS: Overall survival
HR: Hazard ratio
NSCLC: Non-small cell lung cancer
IL6ST: Interleukin 6 Cytokine Family Signal Transducer
GTDC1: Glycosyltransferase Like Domain Containing 1
MAP1B: Microtubule Associated Protein 1B
CPEB3: Cytoplasmic Polyadenylation Element Binding Protein 3
PRKACB: Protein Kinase Camp-Activated Catalytic Subunit Beta
NFIB: Nuclear Factor I B
PURB: Purine Rich Element Binding Protein B
ATP2B1: Atpase Plasma Membrane Ca2 + Transporting 1
ZNF148: Zinc Finger Protein 148
PSD3: Pleckstrin and Sec7 Domain Containing 3
TBC1D15: TBC1 Domain Family Member 15
PURA: Purine Rich Element Binding Protein A
KLF12: KLF Transcription Factor 12
