# Supplementary material for: Identification and bioinformatic characterization of a serum miRNA signature for early detection of laryngeal squamous cell carcinoma
Source: J Transl Med. 2024 Jul 10;22:647. doi: 10.1186/s12967-024-05385-3 (PMC11238506; doi:10.1186/s12967-024-05385-3)
Supplement: Supplementary file 1 — Additional file 1. [file 12967_2024_5385_MOESM1_ESM.docx]

B

A

**Figure S1 A)** miR-223 basal levels in LSCC (FaDu, HEp-2 and HNO-210) and normal (HaCaT) cell lines. **B)** miR-223 expression levels in HNO-210 cells transduced with either miR-223 Mimic or Inhibitor, analysed in comparison with the corresponding Nc. The p-value was calculated by t-test ^*^ p < 0.05, ^***^ p < 0.001


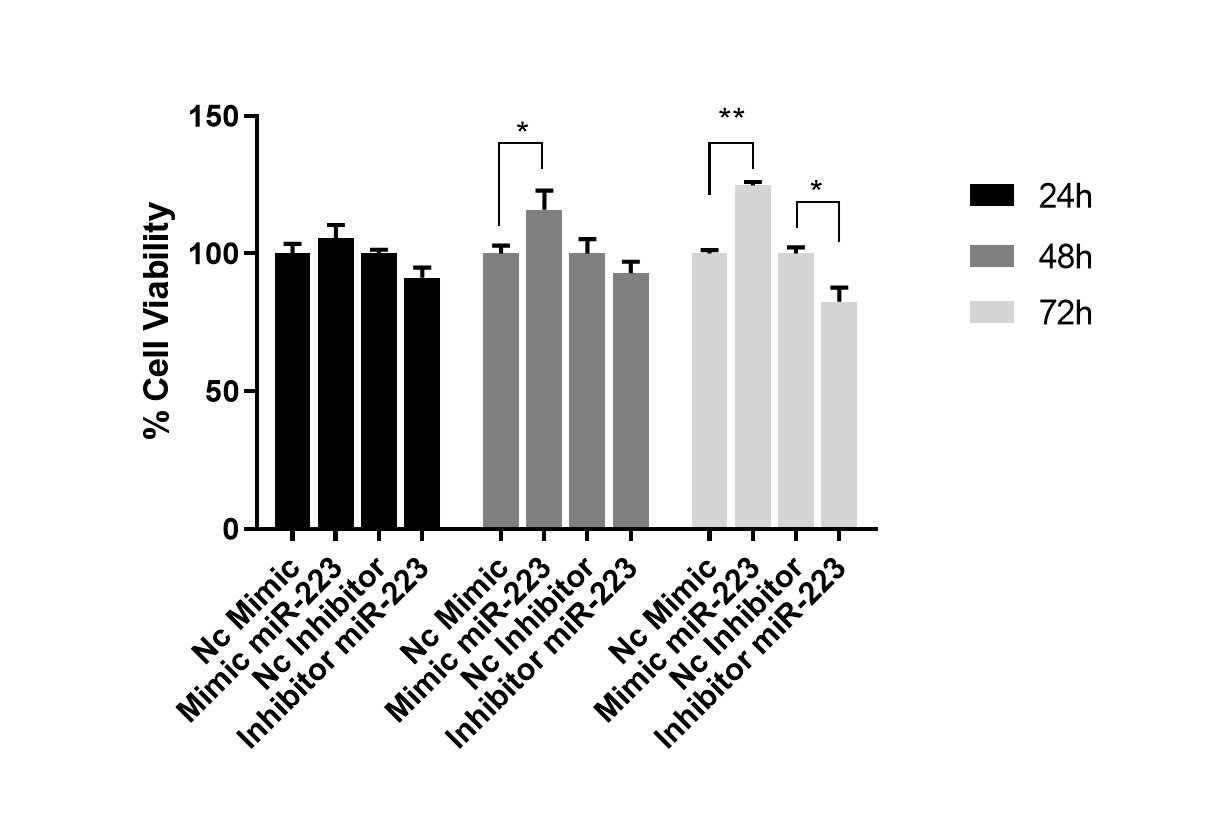


**Figure S2 Ectopic miR-223 Mimic and miR-223 Inhibitor affect HNO-210 cell growth.** Cell growth of HNO-210 cells, transfected with either miR-223 Mimic and miR-223 Inhibitor was evaluated by MTT Assay at 24, 48 and 72h in comparison with the corresponding Nc. The p-value was calculated by t-test ^*^ p < 0.05, ^**^ p < 0.005.


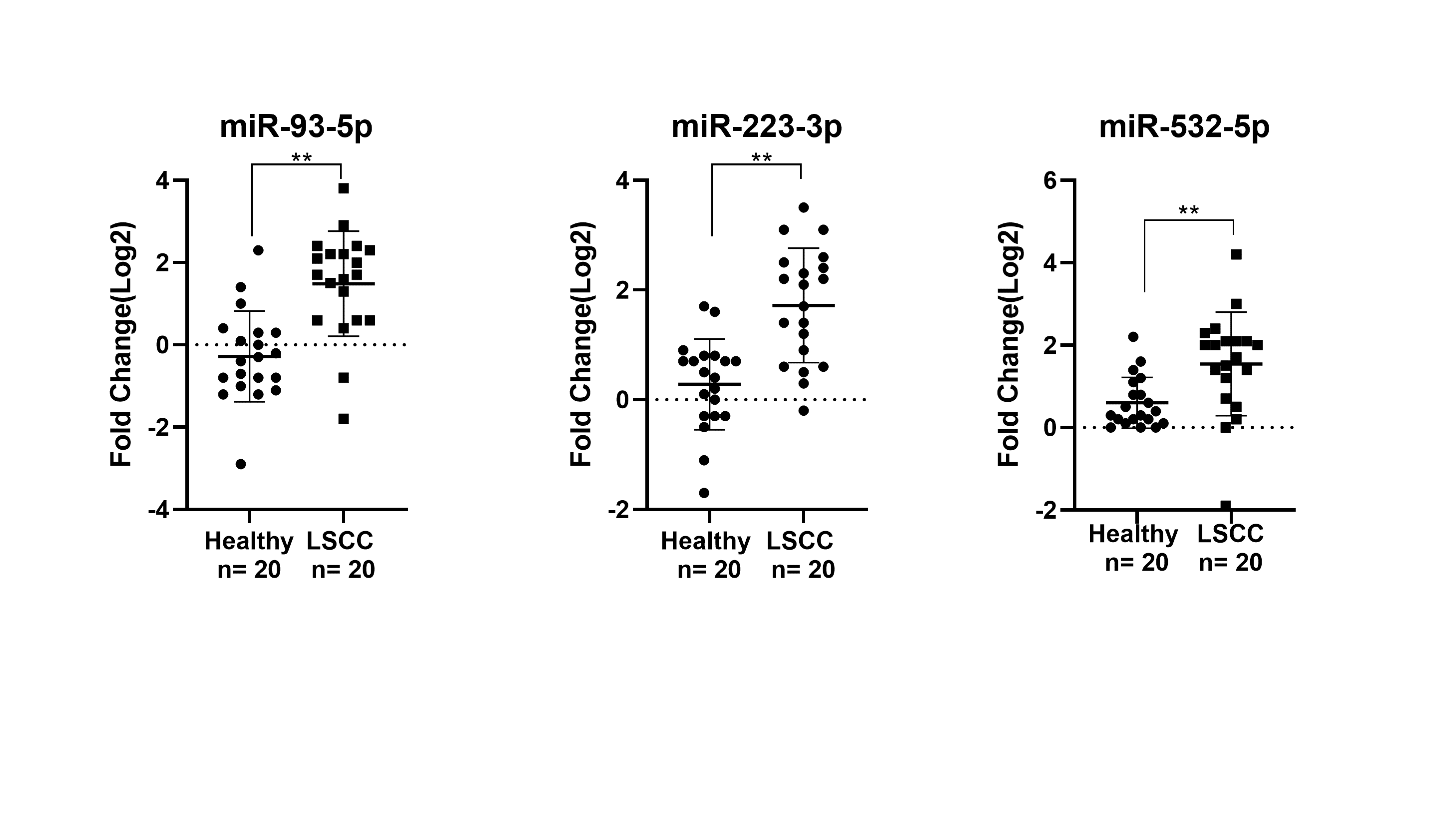


**Figure S3 Serum miR-93, miR-223, and miR-532 expression levels in 20 LSCC patients.** The expression levels of serum miR-93, miR-223, and miR-532 in LSCC patients (n = 20) compared with healthy donors (n = 20), validated by qRT-PCR. Exogenous cel-miR-39 was used as normalizer. The p-value was calculated by t-test **p <0.001.
